# Risk Factors for Age-Related Maculopathy

**DOI:** 10.1155/2009/360764

**Published:** 2009-09-06

**Authors:** Paul P. Connell, Pearse A. Keane, Evelyn C. O'Neill, Rasha W. Altaie, Edward Loane, Kumari Neelam, John M. Nolan, Stephen Beatty

**Affiliations:** ^1^Waterford Regional Hospital, Dunmore Road, Waterford, Ireland; ^2^Macular Pigment Research Group, Waterford Institute of Technology, Waterford, Ireland

## Abstract

Age-related maculopathy (ARM) is the leading cause of blindness in the elderly. Although beneficial therapeutic strategies have recently begun to emerge, much remains unclear regarding the etiopathogenesis of this disorder. Epidemiologic studies have enhanced our understanding of ARM, but the data, often conflicting, has led to difficulties with drawing firm conclusions with respect to risk for this condition. As a consequence, we saw a need to assimilate the published findings with respect to risk factors for ARM, through a review of the literature appraising results from published cross-sectional studies, prospective cohort studies, case series, and case control studies investigating risk for this condition. Our review shows that, to date, and across a spectrum of epidemiologic study designs, only age, cigarette smoking, and family history of ARM have been consistently demonstrated to represent risk for this condition. In addition, genetic studies have recently implicated many genes in the pathogenesis of age-related maculopathy, including Complement Factor H, PLEKHA 1, and LOC387715/HTRA1, demonstrating that environmental and genetic factors are important for the development of ARM suggesting that gene-environment interaction plays an important role in the pathogenesis of this condition.

## 1. Introduction

Age-related macular degeneration (AMD), the late stage of age-related maculopathy (ARM), is the leading cause of blindness in white individuals over 65 years of age in the Western world [1–3]. According to large population-based studies, the prevalence of AMD is 0.2% in persons aged 55 to 64 years, and rises to 13% in individuals 85 years of age or older.

The burden of AMD to individuals and to society is expected to rise as a result of an increase in life expectancy, reduced birth rates, and the consequential demographic shift towards an elderly population [3]. The increasing socio-economic impact of AMD, coupled with its unclear pathogenesis and limited available therapies, has prompted investigators to carry out studies designed to identify risk factors for this condition.

Risk factors for ARM may be classified as modifiable and nonmodifiable. Possible modifiable risk factors for ARM include smoking, body mass index, cumulative sunlight exposure, diet, alcohol consumption, and cardiovascular disease. Possible nonmodifiable risk factors for ARM include: age, family history of ARM (early and/or late), iris color, and refractive error.

Any review of the literature assessing possible risk factors for ARM should comment upon the nature of the designs of the studies cited, and on the relative strengths and limitations of such designs [4]. Cross-sectional study design, in which we take a sample of some narrowly defined population at one point in time, can give information on prevalence of a specific disease, but gives little indication of cause and effect. Furthermore, in certain disease states, the studied end-point may be so infrequent, thus a very large sample size is often necessary. A prospective study is one that is longitudinal in nature with different time points studied. However, such studies are sometimes limited where the population studied is too selective, and hence not representative of the total population. A prospective study design is generally favored over a cross-sectional design when attempting to establish cause and effect [4]. 

Thus, in this article, we review the literature germane to risk for ARM, as determined by studies of various design, including case series, cross-sectional, cohort, and case-control studies (Tables 1 and 2). Further, the relative merits and limitations of varied study designs in investigating risk for a condition such as ARM are also discussed. These studies were chosen for their large sample sizes and the standardized manner in which they have assessed ARM. We also cite nonpopulation-based studies, where necessary, as background material. Of note, the majority of studies relate to white populations [5]. 

Most of the cited studies have utilized a grading system of ARM, consistent with the International Classification and Grading System for ARM and AMD. According to this classification, a diagnosis of early ARM is made in the presence of soft drusen (≥63 *μ*m) within the macular area and/or areas of retinal pigment epithelium (RPE) changes (hyperpigmentation and/or hypopigmentation). The late stage of ARM, known as age-related macular degeneration (AMD), is characterized by at least one of the following features: geographic atrophy, RPE detachment (neovascular or nonneovascular in origin), choroidal neovascularization, with or without its sequelae (hemorrhagic detachment of either the RPE or neurosensory retina, presence of subretinal or sub-RPE hemorrhage or subretinal fibrosis). Geographical atrophy (GA) is defined as a discrete area of hypopigmentation or depigmentation or apparent absence of the RPE in which choroidal vessels are more visible than in surrounding areas, and must be at least 175 *μ*m in diameter. Cases with minimal subretinal fibrosis and widespread surrounding atrophy were also classified as neovascular AMD, because this appearance was considered to indicate previous choroidal neovascularization [5, 6].

In this review article we have, where possible in the context of the grading systems used in the cited studies, distinguished between early ARM and late ARM (AMD). All of the cited studies comment on the prevalence of ARM, defined by the appearance of the designated lesion(s) at the time of the study examination. Where incidence of ARM was studied, it was identified by the appearance of a designated lesion at followup examination a number of years following the baseline examination where no such findings were present.

## 2. Genetic Predisposition

Studying ARM as a genetic disease is difficult due to the inherently age-related nature of the disease [7, 8]. Age-related maculopathy occurs later in life, and therefore only one generation in the affected age range is available for study, as parents may be deceased and the children too young to exhibit this condition. The phenotypic heterogeneity of ARM also presents a challenge, because it is possible that different genes underlie different phenotypes, and therefore genetic studies may fail to identify any one causative gene or region, if all stages of ARM are studied in a collective fashion [9]. Sample size may also be a problem if one specific form of ARM is chosen for analysis, due to the infrequency of the end-point studied. In spite of these difficulties, investigators in recent years have greatly enhanced our understanding of the genetic basis of ARM.

Many human diseases have a genetic basis, mediated through DNA sequence variation [8, 10]. Such DNA alterations may be represented by changes in a single nucleotide or an entire chromosome. Single nucleotide polymorphisms (SNPs) are by far the most common form of DNA sequence variation [8, 10]. Such changes result in altered forms of a particular gene, known as alleles. Different alleles may produce variations in inherited characteristics. These variants may either enhance or reduce an individual's predisposition for developing a particular disease, or they may have no effect on overall disease risk [11].

With regard to ARM, there is now strong evidence supporting the role of genetic background in the development of ARM [8, 10]. The initial evidence supporting the role of heritability in the pathogenesis of ARM was provided by observational familial aggregation studies, twin studies, and segregation analyses [12–15].

**Familial Aggregation Studies**

Familial aggregation studies are designed to identify if the risk for a certain condition is higher in individuals related to the affected individual, when compared to unrelated individuals [16]. One of the first familial aggregation studies undertaken to ascertain the risk of ARM posed by having a positive family history of disease was performed by Seddon et al. [16]. In 1997, they reported that the prevalence of ARM in first-degree relatives of individuals with any form of ARM (atrophic or exudative) was over twice that in individuals who had no family history of disease.

During the baseline examination of the Beaver Dam Eye Study, cross-sectional information was gathered regarding the siblings of participants [17]. Five years later, incident ARM was evaluated for those siblings who had no evidence of the disease at baseline. The odds ratios (ORs) for the sibling developing the lesion, when compared with a control proband, were 8.18 for retinal pigment epithelial hypopigmentation, 3.59 for retinal pigment epithelial hyperpigmentation, and 10.32 for neovascular ARM [17]. 

The Rotterdam Study provides two population-based familial aggregation analyses, which have reported that a positive family history of ARM represents risk for this condition [12, 13]. In the first case control study, first-degree relatives of 87 patients with late ARM were compared with first-degree relatives of 135 control subjects (none of whose first-degree relatives suffered from this condition) [15]. The prevalence of early ARM and late ARM was significantly higher in relatives of patients with late ARM, independent of other risk factors. The second familial aggregation study, within the Rotterdam Eye Study, assessed the extent of heterogeneity of genetic risk of ARM among families [12]. It was reported that considerable differences in risk of ARM heritability between individual families existed, and that the proportion of families at high risk was relatively small.

**Twin Studies**

Several twin studies have also yielded information regarding the nature of heritability of ARM [61, 62]. The first of these, published in 1988, reported on severe ARM in monozygotic twins [61, 62]. This study also demonstrated disease in four of the thirteen twin pairs' siblings [62]. In 1994, Meyers et al. demonstrated a concordance rate of 100% for ARM in 23 monozygotic twin pairs, and a 25% concordance rate in eight dizygotic twin pairs [61, 62]. By comparison, the concordance amongst age- and sex-matched individuals from nontwin subjects ranged from 16 to 25%. Hammond et al. who investigated 226 monozygotic twin pairs and 280 dizygotic twin pairs in a UK study demonstrated an overall prevalence of ARM of 14.6% in their study group, with concordance rates of 37% and 19% for monozygotic and dizygotic twin pairs, respectively [13]. Interestingly, they found that there were differences in the heritability of different phenotypes of ARM, with the fundal appearance of ≥20 hard drusen being dominantly inherited (and 81% genetically determined). In 2005, Seddon et al. reported on a study of 840 elderly male twins, consisting of 210 monozygotic and 181 dizygotic complete twin pairs, and 58 singletons [63]. They devised a model to partition variation in risk for development of ARM into additive genetic, common environment, and unique environment components. Using this model, they reported that genetic factors played a significant role in the etiology of ARM, and accounted for 46–71% of the variation in the overall severity of the disease.

**Genes Involved in ARM Pathogenesis**

The evidence, described earlier, supporting a genetic role in the development of ARM led to a search for a susceptibility gene, or genes, responsible for ARM.

**Complement Factor H**

In early 2005, three separate research groups independently identified a variant in the complement factor H (CFH) gene, known as the Y402H polymorphism, which exerts a strong influence on the risk of developing ARM [64–66]. This variant, in which histidine replaces tyrosine at position 402 of the amino acid sequence of the CFH gene on chromosome 1q31, was associated with odds ratios ranging from 2.45 to 3.33 for all stages of ARM, and odds ratios of 3.45 to 7.4 for late ARM. These studies suggested that almost half of all cases of ARM in older individuals could be attributable to this variant of the CFH gene. Since that report, it has been found that there are five haplotypes at the CFH locus, three of which confer increased risk for ARM, and two of which are protective [65, 66]. The SNP originally associated with increased risk for ARM, responsible for the CFH Y402H variant allele, is rs1061170. A recent article suggests that a 3-SNP haplotype comprising rs1061147, rs1061170, and rs2274700 exhibits the strongest association with advanced ARM, even more so than the rs1061170 SNP alone [66, 67]. It is also interesting to note that polymorphisms of the CFH gene may be more closely associated with risk for geographic atrophy than for neovascular ARM [68]; however, not all studies have reported this preferential association with the GA form of ARM [69, 70].

Interestingly, interaction between tobacco use and the Y402 H gene has been recently reported, although followup studies have not replicated such findings (reported in what follows) [71, 72].

**PLEKHA1 and LOC387715/HTRA1**

Further two reports in 2005 identified other susceptibility loci, on chromosome 10q26, associated with increased risk for ARM [73, 74]. This region of chromosome 10q26 overlaps with three genes: PLEKHA1, LOC387715, and HTRA1, which show significant linkage disequilibrium (LD) with one another [75]. In particular, there is almost complete LD between the LOC387715 and HTRA1 domains [75]. This degree of LD has made it difficult to determine precisely which of these three genes is primarily responsible for the risk of ARM, attributable to this region of chromosome 10q26. However, a recent report by Kanda et al. suggests that a single SNP (rs10490924) that changes the coding sequence of the hypothetical LOC387715 gene can account for all of the association between chromosome 10q26 and risk for ARM [76]. The precise role of the LOC387715 gene has yet to be elucidated, but it is believed to relate to mitochondrial function [76]. Of note, one of the original reports on the LOC387715 gene found that homozygosity for this gene, in combination with homozygosity for the Y402H variant of the CFH gene, confers an odds ratio of 57.6 (95% CI: 37.2–89.0) for the development of ARM, compared to the baseline nonrisk genotype [77]. It is also noteworthy that there appears to be a multiplicative effect on risk for ARM with each additional LOC387715 variant allele, in that heterozygosity confers an odds ratio for development of ARM of 2.83 (95% CI: 1.91–4.20), whereas homozygosity confers an odds ratio of 32.83 (95% CI: 12.53–86.07). However, not all studies have demonstrated such a dramatic multiplicative effect [78].

**Apolipoprotein E**

Apolipoprotein E gene (ApoE) codes for apolipoproteins, which are major transporters of lipid and cholesterol in the nervous system [79–81]. ApoE is polymorphic with three common isoforms: E2, E3, and E4, which are coded for by three separate alleles: *ε*2,  *ε*3, and *ε*4. As a result, six common phenotypes exist: three homozygous phenotypes (*ε*2/*ε*2,  *ε*3/*ε*3,  *ε*4/*ε*4) and three heterozygous phenotypes (*ε*2/*ε*3,  *ε*2/*ε*4,  *ε*3/*ε*4) [82].

Interestingly, ApoE is a ubiquitous component of drusen, and clinical manifestations of retinal degeneration are exhibited in Apolipoprotein E-deficient mice that carry an ApoE gene that has been inactivated by gene targeting [83, 84]. Most studies, but not all, investigating the role of the ApoE gene with respect to the risk for ARM have reported that the *ε*4 allele (which codes for the E4 variant) is less prevalent (thus appearing protective) among sufferers of ARM when compared with a control proband [14, 80, 85–89]. In 1998, a nested case-control study within the Rotterdam Study demonstrated that the *ε*4 allele was associated with decreased risk of ARM, while the *ε*2 allele was associated with a slightly increased risk of ARM [14, 88]. In 2006, the Atherosclerosis Risk in Communities Study found no evidence of a strong association between the ApoE gene and early ARM in middle-aged persons [90]. In 2007, the Cardiovascular Health Study demonstrated that the *ε*2 allele might be associated with an increased risk of developing late ARM in white people aged 65 years and over, and that the *ε*4 allele appears to confer some protection against the development of late ARM (although this association was not statistically significant) [82].

**Other Genes**

Other genes recently discovered to be associated with an increased risk for ARM include the complement factor B gene (BF), the complement component 3 gene (C3), and the complement component 2 gene (C2) [69, 91]. In conjunction with the increased risk of ARM association with the CFH variant gene, these recently discovered complement genes are consistent with the view that inflammation and the control of inflammation are important in the pathogenesis of ARM [92]. Many other candidate genes have been investigated for an association with ARM, including ABCA4, HEMICENTIN-1 (fibulin 6), PON1, ELOVL4, VLDLR, and ACE, with inconsistent results to date [93].

In conclusion, over the past decade, numerous studies have attempted to identify susceptibility genes for AMD. The discovery of a risk variant within the CFH gene and recent findings for loci PLEKHA1/LOC387715, Apo E, and BF/C2 have increased our knowledge particularly in relation to elucidating the complex gene-environment interaction in the pathogenesis of this disease. Future research is likely to clarify the exact role of these genes and identify additional susceptibility genes.

## 3. Cardiovascular Disease

Cardiovascular disease includes coronary heart disease, cerebrovascular disease, and peripheral arterial disease. Atherosclerosis is responsible for the majority of cases of cardiovascular disease [94, 95]. More specifically, atherosclerosis is the most prevalent disease in the modern era, and its thrombotic complications are responsible for an exceedingly high number of deaths and disabilities [94, 96].

As early as 1937, it was hypothesized that ARM may be part of an underlying systemic vascular process and therefore associated with cardiovascular disease [96–99]. Initially, however, epidemiologic studies failed to demonstrate consistent or conclusive results. Interest in the relationship between cardiovascular disease and ARM was renewed following the publication of a work in 1990s that showed a strong positive association between carotid atherosclerosis and ARM [96, 100]. Other studies have since provided further evidence supporting the view that cardiovascular disease is etiologically important for ARM.

### 3.1. Hypothesis/Rationale

The vascular model proposes that the progressive deposition of lipid, seen in atherosclerosis, is the underlying cause of ARM [96, 97, 101]. This deposition of lipid leads to its accumulation in the sclera and in Bruch membrane, with a consequential increase in vascular resistance [101]. This process would then interfere with the metabolism of the RPE and lead to pigmentary abnormalities, drusen formation, and, ultimately, the changes that are represented by the clinical manifestations of ARM [102, 103]. Indeed, a number of in vitro studies support this hypothesis. These include studies which have consistently demonstrated that choroidal blood flow in subjects with ARM is lower than in age-matched controls [98, 100, 104, 105]. Furthermore, accumulation of extracellular cholesterol in Bruch membrane resembles that found in the walls of large systemic arteries [102].

The inflammatory model of cardiovascular disease proposes that local inflammatory responses are important in the etiology of both macular drusen and drusen-like deposits in arterial vessels [98, 106–108]. Several lines of evidence support a role for inflammation in atherogenesis [106, 107]. Chronic inflammatory systemic disorders, including rheumatoid arthritis and anklylosing spondylitis, are associated with an increased incidence of cardiovascular disease [107, 109]. Direct effects and indirect sequelae of systemic inflammation promote atherothrombotic vascular disease. Indeed, an association between cardiovascular disease and systemic markers of inflammation, including C-reactive protein, IL-6, homocysteine, white blood cell count, D-Dimer formation, factor VIII, has been demonstrated [107].

### 3.2. Epidemiologic Studies

Epidemiologic studies investigating a possible association between cardiovascular disease and ARM have typically done so by focusing on three main parameters: (1) clinical evidence of atherosclerosis in subjects with and without ARM; (2) subclinical evidence of atherosclerosis in subjects with and without ARM; (3) risk factors for atherosclerosis in subjects with and without ARM.

#### 3.2.1. Atherosclerosis (Table 4)

In most epidemiological studies, clinically important atherosclerosis was deemed to be present if subjects reported a history of angina, heart attack, or stroke. Atherosclerosis does not correlate well with cardiovascular events, which are largely atherothrombotic [127], and therefore, studies investigating an association between atherosclerosis and ARM must be interpreted with full appreciation of this limitation. Indeed, the majority of studies have failed to demonstrate an association between reported clinically important atherosclerosis and the incidence or prevalence of ARM [4, 18, 125, 128–130].

Three population-based epidemiological studies have investigated whether evidence of subclinical atherosclerosis is associated with risk for ARM, by using noninvasive techniques to measure atherosclerotic vascular changes [123, 129]. Typically, ultrasonography is used to assess the carotid artery for the presence of atherosclerotic plaques, which are defined as focal thickenings of the vessel wall relative to adjacent segments, and composed of calcified or noncalcified components. Aortic plaques were diagnosed by the detection of calcific deposits in the abdominal aorta by plain radiographic films. Peripheral (lower extremity) atherosclerosis was assessed by comparing the blood pressure in the posterior tibial artery to that of the brachial artery (ABI: ankle-brachial index), and an index of <0.9 was considered to indicate peripheral atherosclerosis [131]. 

In 1995, cross-sectional data from the Rotterdam Study indicated that patients with subclinical carotid atherosclerosis or peripheral atherosclerosis exhibited a significantly increased prevalence of late ARM [123, 131–133]. However, this study did not assess early ARM, and one limitation of the analysis rests on the small number of cases of late ARM that was reported. Greater intima-media thickness (another marker of subclinical disease) of the carotid artery and the presence of aortic calcification were also found to be associated with increased risk of incident late ARM in longitudinal analyses of this cohort [134, 135]. Also, in 2003, the Rotterdam Eye Study reported in its prospective arm an association between subclinical atherosclerosis and incident ARM [23, 131].

In 1999, the cross-sectional Atherosclerosis Risk in Communities Study, in fact, demonstrated an association between carotid atherosclerosis and prevalence of early ARM, but not with carotid artery stiffness or pulse pressures (parameters not measured in the Rotterdam Eye Study) [19, 59]. The cross-sectional Cardiovascular Health Study failed to detect an association between early ARM and carotid intima media thickness [57].

The prospective arms of the Beaver Dam Eye Study reported a statistically significant association between a higher pulse pressure and risk for late neovascular ARM (but not atrophic late ARM) [126].

In summary, the results of studies designed to investigate a possible association between atherosclerosis and risk for ARM are inconsistent. However, it is worth bearing in mind that these studies should be interpreted with full appreciation of their limitations, including the poor relationship between atherosclerosis and cardiovascular events, since cardiovascular events were typically used in the reported studies as a presumed indicator of underlying atherosclerosis. This shortcoming needs to be addressed in the design of future studies, if the relationship between atherosclerosis and ARM is to be elucidated.

#### 3.2.2. Cigarette Smoking (Table 3)

Cigarette smoking has been established as an important risk factor in the development of cardiovascular disease [136], and has also been found to be associated with increased risk for ARM [49, 114, 117, 137, 138]. The etiopathogenic mechanism underlying the association between tobacco use and ARM remains unclear. Smoking cigarettes may simply promote vascular changes in the eye in a fashion similar to changes in the systemic circulation and, as such, may simply represent an antecedent common to both atherosclerosis and ARM [96, 97]. Alternatively, a number of other metabolic factors may play a role in the etiopathogenic mechanism underlying the association between tobacco use and the development of ARM, and these include a parallel reduction in circulating levels of antioxidants, coupled with an increased pro-oxidant state caused by cigarette smoke [139]. It has also been demonstrated that there is a relative lack of macular pigment amongst tobacco users [140].

The limitations of studies attempting to investigate the relationship between tobacco use and ARM warrant discussion. First, potential confounders in such studies include age and associations between tobacco use and other health risk behaviours, such as poor diet and increased alcohol consumption. Of the studies discussed in what follows, 15 have been adjusted for some of these potential sources of confounding [49, 52, 72, 110–117, 119, 121, 138, 141]. However, only the 5-year and 10-year followup arms of the Beaver Dam Eye Study adjusted for diet- and alcohol-related factors [110, 116]. It should also be borne in mind that all studies investigating the use of tobacco use and the risk for ARM used subject-reported smoking data, and it is possible that subjects underestimated the extent of their tobacco use. Finally, loss of followup in longitudinal studies may also affect risk factor outcome data, because of smoking-related morbidity and mortality, and therefore represent another potential source of bias.

In epidemiologic studies, smoking history is typically ascertained using an interviewer-administered questionnaire. Participants are usually classified as being nonsmokers, former smokers, or current smokers. In a number of studies, the total pack-years smoked by each participant was also calculated, and this is defined as the number of cigarettes smoked per day divided by 20, and multiplied by the number of years smoked [142].

The relationship between tobacco use and the prevalence and incidence of ARM has been investigated in cross-sectional, prospective cohort and case-control studies (Table 3) [49, 52, 72, 110–114, 116, 117, 119, 121, 138, 141]. Of seventeen studies, 13 have found a statistically significant association between cigarette use and at least one type of ARM, with increased risk of this condition amongst current-smokers and ever-smokers when compared with nonsmokers and never-smokers (relative risk/odds ratio 1.06–4.96) [32, 49, 110–112, 115–119, 121, 141, 143]. A significant positive association between smoking and ARM was observed in six out of seven cross-sectional studies, three of four prospective cohort studies, and four out of six case-control studies. Pooled findings from three well-designed and well-executed cross-sectional studies from Europe, Australia, and USA have also convincingly demonstrated an association between tobacco use and ARM [23, 26]. A prospective design represents the best means for investigating whether smoking leads to the ultimate development of ARM, and such studies have consistently demonstrated an increased incidence of late ARM among smokers, although the association between tobacco use and the incidence of early ARM is less compelling [32, 49, 110–113, 115, 117].

Of note, cigarette use increases the risk for both the neovascular and atrophic forms of late ARM, but neovascular ARM to a greater extent. Of the studies investigating the possible association between cigarette use and late ARM, six out of ten have found that tobacco use represents risk for the development of neovascular late ARM [110, 113, 115, 119–121], and four of five have reported that cigarette use is significantly associated with risk of atrophic late ARM [111, 115, 121, 143]. A smaller number of studies have also confirmed an association between tobacco use and the incidence of both early and late ARM (Table 3) [32, 49, 110–112, 115].

Furthermore, cigarette smoking appears to have a dose-dependent relationship with risk for ARM, reflected in the demonstration that the extent of risk rises with the number of pack years smoked in the Physician's Health Study and in the Nurse's Health Study [117, 118]. To date, eight studies have investigated this dose-response relationship between cigarette use and ARM, and all but one (the 10-year followup arm of the Beaver Dam Eye Study) identified a positive relationship [20, 32, 112, 113, 117, 118, 120, 128]. 

Only one study to date has investigated whether tobacco use influences the age of onset of ARM. In the Blue Mountains Eye study, it was reported that participants who were free of late ARM at baseline but who were current smokers ultimately developed ARM on average 10 years earlier than nonsmokers, who also exhibited no signs of disease at baseline [115].

Finally, there is a growing body of evidence to suggest that cessation of cigarette smoking leads to a subsequent reduction in the risk for ARM. To date, 11 studies have examined the risk of ARM amongst exsmokers, reporting an increased risk of developing ARM when compared to never-smokers, but lower risk when compared to current smokers [20, 32, 111–113, 115, 117, 118, 120, 121, 128]. Indeed, pooled data from the Beaver Dam Eye Study, the Blue Mountains Eye Study, and the Rotterdam Eye Study demonstrated cross-sectionally, and in their extended cohort studies that exsmokers had only a slightly increased risk of ARM when compared to nonsmokers, with even greater risk reduction at extended followup [23, 26]. The Physicians' Health Study evaluated the risk of ARM in exsmokers, taking into account their previous intensity of smoking and the time since cessation. Subjects who smoked >20 cigarettes/day still appeared to have an increased risk of ARM when they stopped smoking >20 years previously [117]. Exsmokers who had smoked <20 cigarettes/day, regardless of when they had stopped smoking, had a similar risk of ARM as did nonsmokers [117].

The results of an association between cigarette smoking and risk for ARM, however, are not entirely consistent, and some studies have failed to identify any association, or have demonstrated only a weak association between tobacco use and ARM [144–146]. Of note, these latter studies should be interpreted with full appreciation of their limitations, including small sample size and the nonprospective nature of the reports.

Finally, although the Beaver Dam Eye Study reported a strong association between smoking and neovascular late ARM at baseline, this association was weaker at the 5-year and 10-year followup examination [20, 147]. However, this latter finding may be attributable to a relatively smaller number of cases at followup (only 56% of baseline subjects were evaluated at the 10-year followup). Indeed, dropout in cohort studies may cause bias towards a null, or even a protective, effect of smoking, if dropout occurred more frequently among smokers, as heavy tobacco use is associated with increased morbidity and mortality. In fact, the effect of such bias is likely to increase with study longevity.

In summary, the current evidence is broadly consistent across a range of populations and study designs. Depending on the type of ARM in question (early, atrophic late ARM, or neovascular late ARM), the risk of developing ARM is two or three times amongst current smokers when compared with never smokers. Three large epidemiologic studies carried out on three different continents, and the pooled data and prospective arms of these studies, provide strong evidence in support of the view that cigarette use is associated with increased risk for ARM [23, 26].

#### 3.2.3. Diabetes Mellitus

Diabetes mellitus is a known risk factor for the development of atherosclerosis [96, 97]. In many epidemiologic studies, self-reporting of diabetes or treatment of diabetes was used to determine whether subjects had this condition, whereas some studies included a random blood glucose measurement. All of these studies have been consistent in their failure to identify an association between diabetes mellitus and prevalent or incident ARM. This finding may appear to be inconsistent with the vascular hypothesis for the pathogenesis of ARM. However, decreased scleral rigidity has been reported in patients with diabetes mellitus, and advocates of the vascular hypothesis for ARM argue that this may have a protective effect for this condition, and, therefore, explain the lack of an association between ARM and diabetes mellitus [101]. Other possible reasons for the lack of an observed association between diabetes mellitus and ARM include selective survival (due to increased and premature mortality rates amongst diabetic subjects), particularly in the context of longitudinal study design. Furthermore, coexisting diabetic maculopathy may lead to an ascertainment bias, related to difficulties in the classification of age-related maculopathy [148].

It is interesting to note that a recent publication on glycaemic index (related to carbohydrate intake) and ARM within the AREDS study concluded that a reduced glycaemic index in nondiabetic individuals may be associated with reduced risk for ARM [149]. This observation is important because hyperglycaemia-mediated damage can occur below the diabetic threshold.

#### 3.2.4. Hypertension (Table 4)

Hypertension is a known risk factor for atherosclerosis [96, 97]. Furthermore, alterations in choroidal blood flow are known to occur in the presence of hypertension, consistent with the view that hypertension may be etiologically important for ARM [150–153]. However, the data from epidemiologic studies is not particularly supportive of this hypothesis. For these studies, subjects were deemed to be hypertensive if systolic blood pressure was ≥160 mm Hg and/or diastolic blood pressure was ≥95 mm Hg. Pulse pressure was taken as the difference between systolic and diastolic blood pressure. In many studies, a history of antihypertensive medication was also recorded for each subject. Data from case-control studies and some population-based studies has shown a positive association between hypertension and the prevalence of both early and late ARM [98, 121, 123–126, 128, 154]. However, a large number of population-based studies have failed to show any significant association between the prevalence of either stage of ARM and hypertension [122, 155].

The association between hypertension and incident ARM has also been investigated, and most studies have failed to identify any significant relationship (Table 4). However, prospective data from the Rotterdam study showed that high systolic blood pressure and/or high pulse pressure was associated with increased risk of all ARM subtypes [124]. The investigators also attempted to answer the question as to whether hypertension is an independent risk factor for ARM or only through its association with atherosclerosis. They found that adjustment for measures of atherosclerosis did not attenuate the observed association between hypertension and ARM. These findings from the Rotterdam Study are consistent with those of the Beaver Dam Eye Study, which showed that an increase in systolic blood pressure between baseline and the five-year followup assessment was associated with an increased risk of incident neovascular AMD at the 10-year followup assessment [125, 126, 128]. Taken together, these studies provide convincing evidence of a mild-to-moderate association between hypertension and ARM. Of note, there is no convincing epidemiologic data that shows a reduction in risk for ARM in subjects taking antihypertensive agents [98].

Hypertensive small vessel disease may lead to focal ischemic changes in the cerebral white matter [60]. The association of white matter lesions in the brain and risk for ARM, as detected by magnetic resonance imaging (MRI), has been investigated in population-based epidemiologic studies [60]. In the Cardiovascular Health Study, MRI-detected cerebral white matter changes were associated with risk of early ARM [57], although this finding was not replicated in the Atherosclerosis Risk in Communities Study [60].

Prolonged hypertension may be associated with changes in the retinal vasculature, such as focal arteriolar narrowing, arteriovenous nipping, as well as retinal arteriolar and venular narrowing [60, 156]. The 5-year and 10-year incidence data from the Blue Mountains Eye Study demonstrates that AV nipping and, to a lesser extent, focal arteriolar narrowing are associated with an increased risk of developing late ARM, thus providing indirect evidence that hypertension may be related to the development of ARM [157, 158]. In contrast, however, the Beijing Eye Study found that retinal vascular abnormalities were not significantly associated with either the prevalence or incidence of early or late ARM [153]. Cross-sectional data from the Beaver Dam Eye Study did not find that smaller retinal arteriolar diameter was associated with any ARM end-point, but was associated with incident RPE depigmentation in the prospective arm of that study (an observation also reported in the Atherosclerosis Risk in Communities Study) [59, 157, 159]. Taken together, the data from these cited studies fails to provide convincing evidence that retinal arteriolar changes are related to risk for ARM. It should be stated, however, that ocular changes secondary to systemic hypertension that are associated with ARM, if any, may well be choroidal rather than retinal in manifestation.

#### 3.2.5. Plasma Lipids (Table 5)

Serum lipids include cholesterol and triglycerides, with cholesterol having multiple subtypes, including low-density lipoprotein (LDL) cholesterol, high-density lipoprotein (HDL) cholesterol, and very low density lipoprotein (VLDL) [164]. Most epidemiologic studies investigating a possible association between serum lipids and ARM have measured LDL cholesterol, HDL cholesterol, and triglyceride. As stated, the progressive deposition of lipid is central to the vascular hypothesis of ARM pathogenesis and, therefore, a relationship between serum cholesterol and ARM would be consistent with such a hypothesis [165, 166].

However, evidence for such an association from epidemiologic studies, to date, has been unimpressive, with few reports of an association between hyperlipidaemia and ARM. Nevertheless, high serum cholesterol was associated with a 2.2-fold increased risk of neovascular ARM in the Eye Disease Case-Control Study [119], and the cross-sectional POLA study also reported an association between the presence of soft drusen and high HDL cholesterol [122]. The prospective arm of the Rotterdam Eye Study reported an increased risk of incident ARM in association with high serum levels of HDL cholesterol, in a dose-dependent manner [161].

However, most epidemiologic studies have failed to detect an association between elevated serum lipid levels and late ARM. Moreover, some studies have shown that high LDL cholesterol and low HDL cholesterol were actually protective for prevalent and incident ARM (Table 5), with the pooled cross-sectional data from the Beaver Dam Eye Study, the Blue Mountains Eye Study, and the Rotterdam Eye Study reporting a nonsignificant reduction in risk for neovascular and atrophic late ARM in association with increasing cholesterol levels [23, 26, 167]. To explain these seemingly contradictory findings, the investigators speculated that high plasma cholesterol levels might lead to downregulation of LDL receptors in the retinal pigment epithelium (RPE), with a consequential protective effect [23, 26, 167]. 

Epidemiologic studies and clinical trials have investigated the relationship between cholesterol-lowering agents (statins) and ARM, but until recently, no association between the use of these medications and the incidence or progression of ARM has been reported [125, 128, 160, 162, 167–169]. For example, prospective data from the 15-year followup examination of the Beaver Dam Eye Study reported no protective effect with statin use, as assessed at the 10-year examination, with respect to incident early or late ARM, or with respect to progression to late ARM [18, 19, 170]. However, in the latest analysis of the Blue Mountains Eye Study, users of statin at baseline and at 5-year followup had a 67% lower risk of developing indistinct soft drusen at the 10-year followup examination, and this differed from the prospective 5-year analysis of the same study where no such association was reported [169].

Any attempt to reconcile the discrepancy of the results between two similarly and well-designed prospective arms of population-based studies must take account of the differences in baseline findings between the Beaver Dam Eye Study (BDES) and the Blue Mountain Eye Study (BMES). For example, the ratio of statin users to nonusers was 1 : 14 and 1 : 4 in the BMES and the BDES, respectively [18, 19, 169, 170]. Furthermore, the conversion to late ARM was very low in both studies, and the BMES and the BDES only had sufficient power (90% with an *α*-level of 0.5) to detect significant relative risk of 0.27 or 0.5, respectively [18, 19, 169, 170].

Attempting to identify the causal or protective effect from data generated from a population-based study can be complex, and warrants discussion. Cross-sectional studies, in which the disease and exposure are measured at the same time, inherently lack the ability to detect a true temporal association. Cohort studies, such as the prospective arms of the Beaver Dam Eye Study, the Blue Mountains Eye Study, and the Rotterdam Eye Study, allow for such inferences. However, and in the context of an infrequent risk factor or an infrequent end-point (such as conversion to neovascular ARM) prospective population-based studies may have limited power to detect an association. Therefore, the question remained unanswered is whether statin use is protective for development, or progression, of ARM. However, further followup of large cohorts with increasing numbers taking statins should enhance our understanding of the protective role, if any, of statins for ARM. Nevertheless, and even with more prolonged followup, difficulties arising from unmeasured confounding, selective survival, and other sources of bias will mean that the result of such studies should be interpreted with appropriate caution.

#### 3.2.6. Obesity (Table 6)

Obesity is an established risk factor for cardiovascular disease, and many studies have shown that obesity is a risk factor for hypertension and lipid abnormalities, and, therefore, predisposes to atherosclerosis [96, 97]. Therefore, obesity may be a risk-factor for ARM only insofar as it is an antecedent common to both cardiovascular disease and ARM. Obese people may also have a decreased dietary intake of important nutrients that are believed to protect against ARM [171]. Alternatively, increased body fat may impair antioxidant defense mechanisms within the retina. For example, lutein and zeaxanthin are carotenoids found in the retina, where they are known collectively as macular pigment, and are believed to protect the central retina from oxidative damage [172–175]. However, adipose tissue is a major storage organ for these carotenoids, and it has been shown that there is a relative lack of macular pigment in obese subjects, thus attenuating the macula's natural antioxidant defense mechanism [171, 176]. Furthermore, there is a growing body of evidence implicating inflammatory mechanisms in the pathogenesis of ARM, and such processes are also linked with obesity (discussed in what follows) [177].

Body mass index (BMI) is defined as the body weight in kilograms divided by the height in meters squared, and is the most commonly used measure of obesity in epidemiologic studies [177–179]. Individuals with a BMI > 30 are categorized as obese. In 2003, evidence from a large cohort study demonstrated that a higher BMI was significantly associated with risk for progression from early-to-late ARM [177–179].

Evidence to date from other studies is less convincing (Table 6). In the cross-sectional arm of the Beaver Dam Eye Study, BMI was a significant risk factor for prevalent pigmentary abnormalities in women, but not for prevalent late ARM [180]. The prospective arm of the Beaver Dam Eye Study also showed that, at five years, incident retinal pigmentary changes (but not other ARM end-points) were more common in obese subjects than in nonobese subjects [181].

In the cross-sectional POLA study, participants with a BMI > 30 showed a two-fold increased risk of late ARM, when compared to subjects with a BMI < 25, and it was also observed that participants with a BMI > 30 had an increased risk of early ARM in the form of pigmentary changes at the macula [163].

In the cross-sectional arm of the Blue Mountains Eye Study, a high BMI and a low BMI were both associated with an increased risk of prevalent early ARM [182]. However, it should be noted that pooled data from the prospective arms of the Beaver Dam Eye Study, the Blue Mountains Eye Study, and the Rotterdam Study failed to detect an association between increased BMI and incident ARM [23, 26].

Two recent, nonpopulation-based, prospective studies have reported a two-fold increased risk of incident ARM in obese subjects. The Physicians' Health Study, a prospective study of male physicians in USA, reported a two-fold increased incidence of early ARM amongst obese subjects [117]. However, this same study also reported that the leanest men (BMI < 22) were also at increased risk of early ARM, independent of other potential confounding variables, thus, suggesting a J-shaped association of BMI and risk for ARM. However, the prospective Hisayama study failed to detect such any association between BMI and ARM [50, 51].

In conclusion, conclusive evidence that BMI represent risk for ARM is still lacking. However, there appears to be a growing body of evidence in support of the view that obesity is an important determinant for the development and/or progression of ARM.

#### 3.2.7. Physical Inactivity and Inflammation

Obesity may be a marker of lack of physical activity, and such a lack of physical activity is associated with increased risk of several factors that predispose to cardiovascular disease, including hypertension, weight, and lipid profile [107]. Indeed, engaging in regular physical activity reduces the risk of developing cardiovascular disease [183]. Following acute exercise, there is a transient increase in the circulating levels of anti-inflammatory cytokines but with more prolonged exercise, a reduction in proinflammatory cytokines has been demonstrated [184]. Limited studies in humans and more comprehensive assessments in animals have confirmed the athero-protective effect of exercise [185, 186]. The Beaver Dam Eye Study prospectively investigated whether physical activity was related to the incidence of ARM, and found that after controlling for potentially confounding variables, people with an active lifestyle were at reduced risk of developing neovascular late ARM, when compared with people with more sedentary lifestyles [187].

#### 3.2.8. Female Sex Hormones (Table 7)

There is a growing debate amongst epidemiologists about the role of female sex hormones in the etiopathogenesis of cardiovascular disease. The observation that the risk of cardiovascular disease is lower amongst women than amongst men before the menopause, but rises in postmenopausal women, is well documented. Indeed, observational studies have reported that hormone replacement therapy, in the form of exogenous estrogen, is protective against cardiovascular disease [191, 197–199].

A number of mechanisms have been put forward to explain the putative protective effect of female sex hormones for cardiovascular disease [200]. First, estrogen may lead to favorable alterations in serum lipids, fibrinogen, or plasminogen levels, and may exert antioxidant properties [200]. Furthermore, changes in BMI and blood pressure are also observed following the menopause, and such changes may be etiologically important for ARM [191, 197, 198].

However, results from recent randomized controlled studies, including the Heart and Estrogen/Progestin Replacement Study (HERS) [201] and the Womens' Health Initiative (WHI) [202], suggest a more complex relationship between hormone replacement therapy and cardiovascular disease, with results which differ from those of the observational studies cited earlier (which are based on the time from menopause to initiation of hormone replacement therapy). Indeed, the HERS, and subsequently the estrogen/progestin arm of WHI, suggested that HRT may be associated with an increased risk for cardiovascular in postmenopausal women. However, it must be stated that these results have not been confirmed by the more recent report of the WHI on estrogen only, where a protective effect of this hormone on cardiovascular endpoints in young postmenopausal women was demonstrated [203]. It appears, therefore, that time to initiation of HRT, and the type of therapy, may be important when investigating the possible role of female sex hormones in cardiovascular disease, and possibly also in ARM [201, 202].

Epidemiologic studies have used a number of ways to measure exposure to estrogen (Table 7). Exposure to endogenous estrogen is related to the age at menarche, age at menopause, and the number of pregnancies. Exposure to exogenous estrogen is related to the use of hormone replacement therapy and to the use of oral contraceptives.

The cross-sectional arm of the Beaver Dam Eye Study found that high parity (i.e., decreased exposure to endogenous estrogen) was associated with an increased prevalence of early ARM [180, 181]. The same study also found that HRT (exogenous estrogen) usage was associated with a decreased risk of late ARM [180, 181]. The cross-sectional arm of the Blue Mountains Eye Study found that increasing number of years from menarche to menopause (i.e., increased endogenous estrogen exposure) was associated with a lower prevalence of early ARM [180, 181, 188]. The cross-sectional Los Angeles Latino Eye Study reported that exogenous estrogen had a protective effect against the development of early ARM [114]. In contrast with the earlier findings, the cross-sectional data from the POLA Study [189], Salisbury Eye Evaluation Project [190], and the Aravind Comprehensive Eye Survey [191] failed to identify any significant protective effect associated with exposure to endogenous estrogen.

Incidence data from prospective studies is similarly unconvincing. Five-year incident data from the Beaver Dam Eye Study provided little evidence of any association between exposure to estrogen and incident ARM [181]. Interestingly, the Rotterdam Study found that increased number of years from menarche to menopause (i.e., increased exposure to endogenous estrogen) was directly related to incident geographic atrophy [192]. However, when this data was pooled with findings from the Beaver Dam Eye Study and the Blue Mountains Eye Study, no overall association between estrogen exposure and incident ARM could be reported [23, 26].

In summary, reports of studies designed to investigate an association between exposure to estrogen (whether exogenous or endogenous) and ARM are inconsistent. Longitudinal analyses of prospective cohorts are required to enhance our understanding of the association, if any, between estrogen and risk for ARM. Well-designed, randomized controlled studies are needed to investigate the putative beneficial effect of HRT on incidence and/or progression of ARM.

#### 3.2.9. Other

A number of emerging risk factors for atherosclerosis have recently been identified. These “novel” risk factors include lipid-related factors, inflammatory markers, and infectious agents. [143, 154, 204]. Of note, some of these risk factors are of interest to researchers investigating risk for ARM in a way that is beyond the risk that these variables represent for cardiovascular disease (e.g., inflammatory markers).

Apolipoproteins and lipoproteins are essential in the transport of cholesterol and lipids. Apolipoprotein E has three common alleles (*ϵ*2, *ϵ*3, *ϵ*4), which influence total and LDL cholesterol levels. The association between the apolipoprotein E gene and ARM has been discussed earlier in this review.

Inflammatory markers that have been implicated as risk factors for cardiovascular disease include C-reactive protein, interleukins, serum amyloid A, vascular and cellular adhesion molecules, and white blood cell count [205]. Many of these markers have been investigated for an association with ARM in population-based studies, and the findings are presented elsewhere in this review (reported in what follows).

Homocysteine is an amino acid, which is formed as a by-product of the metabolism of the essential amino acid methionine [154]. Elevated levels of homocysteine are associated with an increased risk of cardiovascular disease. Folate, cobalamin (vitamin B12), and pyridoxine (vitamin B6) are involved in the metabolism of homocysteine as cofactors, and low levels of these vitamins may lead to high levels of homocysteine. However, and with respect to ARM, data from population-based studies is limited. In the National Health and Nutrition Examination Survey III, analysis of cross-sectional data revealed that total serum homocysteine, red blood cell folate, and serum cyanocobalamin were unrelated to ARM [55]. In 2007, the Blue Mountains Eye Study found that high levels of serum homocysteine and low levels of serum vitamin B12 were independently associated with an increased risk of late ARM [206].

Infectious agents, such as cytomegalovirus, *Chlamydia pneumoniae*, and *Helicobacter pylori* have been putatively linked to risk for cardiovascular disease [207–209]. A single study demonstrated a significant association between high cytomegalovirus IgG titers and neovascular AMD, but no association between *Helicobacter pylori* IgG titers and ARM [209]. The association between ARM and either cytomegalovirus or *Helicobacter pylori* has not been examined, thus far, in population-based epidemiologic studies. Research has been carried out on the relationship between *Chlamydia pneumoniae* and ARM, although the findings have been inconsistent [207, 210]. The Cardiovascular and Age-Related Maculopathy (CHARM) Study demonstrated that participants with high titers of antibodies to *Chlamydia pneumoniae* exhibited an increased risk of ARM progression [58].

## 4. Indicators of Inflammation (Table 8)

It has also been postulated that ARM may represent an ocular manifestation of chronic inflammatory processes [211]. This hypothesis has recently been given fresh impetus by the discovery that patients with a certain variant of the complement factor H gene (Y402H) are at significantly increased risk of developing ARM [65].

### 4.1. Hypothesis/Rationale

The inflammatory model proposes that some form of tissue injury leads to a localized inflammatory response in the retina, involving human leukocyte antigen (HLA) and the complement system [65, 106, 211]. This inflammatory response leads to drusen formation and alteration of the extracellular matrix, which, in turn, leads to altered RPE-choriocapillaris behavior and, ultimately, to choroidal neovascularization and other changes seen in late ARM [106, 211, 212]. Indeed, a number of in vitro studies provide evidence in support of this view. For example, the cellular and molecular constituents of drusen have been analyzed in detail [102, 103, 106, 213], and have been shown to contain proteins associated with immune-mediated processes and inflammation, such as complement components, immunoglobulins, and anaphylatoxins. Furthermore, histological studies have consistently demonstrated chronic inflammatory cells within retinas afflicted with ARM. It is believed that these inflammatory cells may damage tissue by release of proteolytic enzymes, oxidants, and toxic oxygen compounds. 

The human immune system contains both innate and adaptive components [102]. The innate immune system includes complement, macrophages, and leukocytes. The complement system is activated by foreign proteins or damaged cells leading to their destruction by host defenses. C3b is a component of complement that is deposited on both host and foreign cells [214]. Complement factor H (CFH) binds and inactivates C3b deposited on intact host cells, thereby preventing their destruction, and, thus, playing a critical role in the regulation of this inflammatory process. Recent genome-wide linkage analyses have identified a gene locus for ARM on Chromosome 1, and case-control studies went further, identifying CFH as the responsible gene [64, 65, 215, 216]. This gene has many polymorphisms that relate to ARM. Of these, the Y402H variant, which is located within a binding site for C-Reactive Protein (CRP), has the strongest association with ARM [64, 65, 215, 216]. This finding enhances our current understanding of ARM pathogenesis, and is supportive of the view that inflammatory changes are etiologically important for this condition.

### 4.2. Epidemiologic Studies

A number of case-control and cohort studies have provided evidence in support of the view that inflammatory changes are etiologically important for ARM. Until recently, however, the evidence from large population-based epidemiological studies has been less consistent in this regard (Table 8). These studies have tended to look at associations between ARM and systemic diseases with inflammatory components, as well as markers of systemic inflammation. In 2003, the Beaver Dam Eye Study published data that revealed a modest association between the 10-year incidence of ARM and gout and pulmonary emphysema, independent of cigarette smoking and other risk factors [194]. Of note, however, aspirin and other nonsteroidal anti-inflammatory agents have not been found to be associated with risk reduction for ARM [52, 53].

The Beaver Dam Eye Study demonstrated an association between the 10-year incidence of ARM and high white blood cell count and with low serum albumin [20, 128, 194, 195], but this finding has not been consistent.

Plasma fibrinogen is a well-established marker of systemic inflammation, and elevated fibrinogen levels are seen in association with arthritis, diabetes mellitus, and certain cardiovascular risk factors [193]. The Cardiovascular Health Study failed to detect an association between serum fibrinogen levels and the prevalence of early ARM [193]. However, the Blue Mountains Eye Study demonstrated an association between elevated fibrinogen levels and the prevalence of late ARM [217].

In 2004, Seddon et al. demonstrated raised CRP levels in association with intermediate and advanced ARM, when compared with a control proband [218]. The prospective followup of CRP levels with respect to ARM, published in 2005 (by the same authors) demonstrated that both CRP and IL-6 were significantly and independently related to progression of ARM, after adjusting for potential confounders [219]. Indeed, subjects in the highest quartile of CRP had a two-fold greater risk of ARM progression than subjects in the lowest quartile, thus corroborating the conclusions of the previous cross-sectional study [218, 219]. However, an association between CRP and ARM was not observed in the cross-sectional data from either the Cardiovascular Health Study or the Beaver Dam Eye Study [57, 193].

More recent epidemiologic studies have been able to investigate the role of complement factor H Y402H polymorphism with respect to ARM. The Rotterdam Study tested for the CFH Y402H polymorphism in a total of 5681 individuals (11363 alleles), and identified this polymorphism in 36.2% [220, 221]. It was reported that the risk of developing late ARM by age 95 years was 48.3% and 42.6% for homozygotes and heterozygotes, respectively, with this polymorphism, and this compares with only 21.9% for noncarriers. In that study, erythrocyte sedimentation rate (ESR) and C-reactive protein (CRP) levels were also measured, and it was found that both of these markers of systemic inflammation were associated with increased risk of ARM in carriers of the CFH Y402H polymorphism, but that neither of these markers represented risk for developing ARM in noncarriers [220, 221]. This novel and interesting finding may explain the inconsistent results with respect to the relationship between these markers of systemic inflammation and ARM in previous studies. Indeed, it was this potential source of confounding that prompted the authors to, therefore, examine the CRP gene in relation to ARM, and it was found that CRP haplotypes that increase serum levels of CRP are associated with significantly enhanced effects of CFH Y402H polymorphism [220]. Also, and interestingly, the same investigators found that smoking cigarettes increased the risk of early and late ARM by a factor of 3.3, and that homozygosity for the CFH Y402H polymorphism increased the risk by a factor of 12.5; however, the combination of both these risk factors resulted in an increased cumulative risk of ARM by a factor of 34 [72, 221]. In other words, and because tobacco use has been shown to activate the complement system, smoking cigarettes may accentuate and facilitate the deleterious effects of inflammatory changes with respect to ARM in an individual with the CFH Y402H polymorphism. This represents an example of an interaction between genetic background and environment to promote a pathologic process [72, 221]. 

Other, and more novel, markers for inflammation have also been examined with respect to ARM. In two samples of participants from the Beaver Dam Eye Study, no association was found between serum amyloid A, interleukin-6, or tumor necrosis factor and the prevalence or incidence of ARM [194, 195]. This study also examined markers of endothelial cell dysfunction (intercellular adhesion molecule-1 (ICAM-1) and E-selectin), and failed to detect an association between either of these parameters and the risk for ARM.

## 5. Indicators of Oxidative Stress

Animals release energy from dietary carbohydrates, proteins, and lipids by cellular oxidative processes. These processes include the tricarboxylic acid (TCA) cycle and oxidative phosphorylation [173, 222]. Cellular damage arising from oxidative processes appears to play a role in physiological and pathological aging. The free radical theory of aging proposes that aging and age-related disorders are the result of cumulative damage arising from reactions involving reactive oxygen intermediates [173, 222, 223]. 

Reactive oxygen intermediates (ROIs) is a term used to describe free radicals, hydrogen peroxide, and singlet oxygen. These molecules are produced in the cell as by-products of oxidative processes, and result in cytotoxic oxidative chain reactions. An increase in the production of ROIs is seen in association with aging, and in association with inflammation, cigarette smoking, and irradiation [173, 222, 223]. 

Cells have multiple mechanisms to protect against the effects of oxygen toxicity [173]. These include DNA repair mechanisms and the separation of ROIs from cellular components that are susceptible to oxidative damage. Also, several enzymes have antioxidant properties, and these include glutathione peroxidase, catalase, and superoxide dismutase [224, 225]. Numerous vitamins also have antioxidant properties, reacting directly and nonenzymatically with ROIs, yielding harmless products [226].

### 5.1. Hypothesis/Rationale

Oxidative processes occur in the retina, and it is postulated that they are etiologically important in the pathogenesis of ARM [173]. For a number of reasons, the retina represents an ideal environment for the generation of, and damage by, ROIs [173, 226, 227]. First, the retina has a much greater consumption of oxygen than other tissues. Second, the retina is subjected to a lifetime of cumulative light exposure, and thus high levels of cumulative irradiation [173, 226, 227]. Third, the outer segments of photoreceptors are rich in polyunsaturated fatty acids and these molecules are especially susceptible to oxidative damage [224, 225]. Fourth, the neurosensory retina and RPE contain an abundance of photosensitizers, which generate oxidative chemical reactions in response to light [173]. Finally, phagocytosis by the RPE results in the production of hydrogen peroxide [228].

In other words, it appears that oxidative damage of the retina may represent a common pathway, which is important for the development of ARM, and which is not inconsistent with other hypothesized etiologies and/or risk-factors for ARM, including cumulative light damage, inflammatory processes, hemodynamic abnormalities, and genetic predisposition.

### 5.2. Epidemiologic Studies

Although much of the work on oxidative processes in relation to ARM has been laboratory-based, there is now a growing number of epidemiologic studies investigating the role, if any, of oxidative processes in the pathogenesis of ARM.

Such epidemiologic studies tend to investigate parallels between the prevalence (and/or incidence) of ARM and antioxidant or pro-oxidant status [229].

#### 5.2.1. Antioxidant Status

Antioxidant status is assessed by evaluating defense mechanisms against oxidative stress. Such measures may include dietary intake of relevant antioxidants, or serum or tissue (e.g., macular pigment optical density) levels of such antioxidants [225, 229–231].

**(a) Vitamin C (Table 9)**

Vitamin C (ascorbic acid) is thought to be essential for protection against disease processes caused by oxidative stress. It is the most effective aqueous phase antioxidant in human blood [232]. Foods rich in vitamin C include citrus fruits/juices, green peppers, broccoli, and potatoes [232]. In 1988, cross-sectional data from the National Health and Nutrition Examination Survey (NHANES) I reported that the consumption of foods rich in vitamin C was negatively associated with the prevalence of ARM (Table 8) [54]. However, after adjusting for demographic and medical factors, this association was no longer present [54].

The Age-Related Eye Disease Study (AREDS) provided evidence that, in addition to zinc, antioxidant supplementation may slow ARM progression in relatively advanced early ARM cases [121, 175]. In that study, trial participants who received a daily high-dose supplement of vitamins C and E and B-carotene had an approximately 25% reduced risk of progression from relatively advanced early cases of ARM to late stage disease (OR 0.72). Vitamin C in isolation was not examined. Furthermore, this study did not address the question of primary prevention in those with no signs of ARM.

Cross-sectional data from the Beaver Dam Eye Study also showed that a high dietary intake of vitamin C was negatively associated with early ARM, but this finding was not statistically significant [229, 237]. In 1999, cross-sectional data from the Blue Mountains Eye Study reported no association between vitamin C and the prevalence of early or late ARM [229, 233], and the investigators subsequently reported, in prospective arms of these studies, that high intake of vitamin C from diet and/or supplements was neither associated with an increased or decreased risk of incident early ARM [226, 229]. Finally, in the prospective arm of the Rotterdam Eye Study, it was shown that an above-median intake of vitamin C, in combination with vitamin E, beta-carotene, and zinc, was associated with a significant 35% reduction in risk for incident ARM [229, 235]. However, it should be noted that such a protective effect was not detected with dietary intake of vitamin C in isolation of the other nutrients mentioned earlier, consistent with the view that antioxidants work synergistically and that a broad spectrum of antioxidant intake is important to protect against oxidative stress. 

Serum levels of vitamin C have also been examined in relation to risk for ARM. Cross-sectional data from the Baltimore Longitudinal Study of Aging reported a nonsignificant protective effect associated with high levels of plasma vitamin C, but this finding was not replicated in the POLA study [29, 229, 236]. 

In summary the relationship between vitamin C and ARM remains unclear. The majority of studies have failed to demonstrate a clear association between either dietary intake or serum levels of vitamin C and ARM (OR 0.6–1.15).

**(b) Vitamin E (Table 10)**

Vitamin E is the major antioxidant of cellular membranes [229, 230, 238]. It exists in four common forms, including *α*-tocopherol, *β*-tocopherol, *γ*-tocopherol, and *δ*-tocopherol. *α*-Tocopherol is predominant in human retina and plasma [238, 239], and, within the retina, is located in the rod outer segments and in the RPE, and the concentrations within these tissues are very sensitive to dietary intake of this vitamin [238, 240, 241]. Food rich in vitamin E includes whole grains, vegetable oil, eggs, and nuts [238]. Dietary intake of vitamin E is difficult to estimate because the long-term consumption of oils is difficult to determine by questionnaire.

The Beaver Dam Eye Study showed that the prevalence of early ARM was lower for people in the highest versus lowest quintiles for dietary intake of vitamin E (Table 9) [237], although this association did not reach statistical significance. The Beaver Dam Eye Study subsequently looked at the incidence data, and found that high dietary intake of vitamin E was associated with a decreased incidence of large drusen but that, overall, it was not protective for early ARM [229]. More recently, the Rotterdam Study published strong evidence that a high dietary intake of vitamin E was associated with a lower risk of incident ARM [229, 235] and reported that exclusion of supplement users did not attenuate their findings.

Less evidence has been published on the association, if any, between serum levels of vitamin E and ARM. Vitamin E is transported by lipoproteins, and it is increasingly recognized that plasma *α*-tocopherol levels should be expressed in terms of its concentration within these lipoproteins [229, 235]. A nested case-control study within the Beaver Dam Eye Study showed no statistically significant association between average serum levels of vitamin E and ARM [229]. Examination of samples from the Blue Mountains Eye Study also failed to detect an association between serum vitamin E and ARM [229, 242]. The POLA study carried out the most comprehensive examination of the link between vitamin E and ARM [29, 229, 236], and the investigators found that plasma *α*-tocopherol levels showed a weak negative association with the prevalence of late ARM. Furthermore, the POLA investigators were the first to assess the lipid-standardized levels of *α*-tocopherol, and these measures also showed a significant negative association with late ARM [29, 229, 236]. Similarly, cross-sectional data from the Baltimore Longitudinal study of Aging demonstrated a significantly lower risk of ARM in subjects with the highest versus lowest plasma levels of vitamin E [231]. However, vitamin E intervention studies have failed to detect a protective effect of vitamin E supplementation on the development or progression of ARM [229, 243, 244].

Therefore, the body of evidence that vitamin E is protective for ARM remains inconsistent.

**(c) Vitamin A (Table 11)**

Vitamin A (retinol) is vital for the regeneration of rhodopsin in the retina, and is therefore essential for vision [54, 229]. There is some evidence to suggest that retinol has antioxidant activity in photoreceptor cells, and that it is also involved in the repair of cells that have been oxidatively damaged. NHANES I provided evidence that dietary intake of foods rich in vitamin A were negatively associated with the prevalence of ARM, even after adjustment for demographic and medical factors (Table 10) [54, 229], but this finding has not been corroborated by other large population-based studies [233, 236]. The Blue Mountains Eye Study failed to detect an association between dietary intake of vitamin A and either the prevalence or incidence of ARM [229, 233]. Furthermore, the POLA study also failed to detect an association between plasma retinol and ARM [29, 229, 236].

**(d) Carotenoids (Table 12)**

Carotenoids are naturally occurring pigments [140]. Some carotenoids can be converted to vitamin A and are, therefore, classed as provitamin A cartenoids. Carotenoids also have antioxidant properties, and act synergistically with other antioxidants [139, 140, 173, 247]. Thirty four carotenoids have been identified in serum and, of these, only lutein, zeaxanthin, and meso-zeaxanthin are found in the human retina, where they are collectively referred to as macular pigment [139, 140, 173, 247–249]. Macular pigment is entirely of dietary origin, and can be augmented with appropriate dietary modification and/or supplementation [250]. As well as direct antioxidant effects, macular pigment is also believed to limit retinal oxidative damage by filtering out blue light [251]. The macular carotenoids are found in high concentrations in certain foods, such as carrots, kale, and spinach [250].

In the Beaver Dam Eye Study, dietary intake of carotenoids was unrelated to the prevalence of early or late ARM (Table 11) [252]. However, further work from the Beaver Dam Eye Study showed an inverse association between dietary intake of pro-vitamin A carotenoids and the incidence of large drusen [252]. Of note, the Blue Mountains Eye Study failed to detect an association between dietary intake of *α* and *β*-carotenes and the prevalence or incidence of ARM [233, 242]. More recently, the Rotterdam Study showed that a high intake of beta-carotene, in combination with vitamin C, vitamin E, and zinc, was associated with a significantly reduced risk of incident ARM [235]. However, in that study, the associations between ARM and either beta-carotene, lutein, or zeaxanthin in isolation were not found to be significant.

However, the macular carotenoids are likely to be more relevant to risk for ARM than B carotene, especially since B-carotene is not found in the human neurosensory retina. Foods that are rich in lutein and zeaxanthin include leafy green vegetables, corn, green peppers, and egg yolks [140]. Data from the NHANES III showed that in younger age groups, higher levels of lutein and zeaxanthin in the diet were related to lower risk of retinal pigmentary abnormalities [245]. Overall in this study, however, no convincing evidence of an inverse relationship between dietary intake of lutein and zeaxanthin and the risk of ARM was detected. Nevertheless, the AREDS study provided evidence that dietary intake of lutein and zeaxanthin was inversely associated with neovascular ARM (OR 0.65), geographical atrophic changes (OR 0.45) and large or extensive intermediate drusen (OR 0.73) [121, 175].

Serum levels of *α*- and *β*-carotene, *γ*-cryptoxanthin, and lycopene, as measured in subsamples of the Beaver Dam Eye Study and Blue Mountains Eye Study, failed to demonstrate any consistent associations with ARM [229]. Of note, in 2001, the NHANES III reported no relationship between serum lutein (or zeaxanthin) and any form of ARM [229, 245]. However, until recently, lutein and zeaxanthin in the plasma could not be easily separated. In 2006, the POLA study demonstrated that high plasma zeaxanthin was associated with a markedly reduced risk of ARM [229, 246]. High plasma lutein, total lutein, and zeaxanthin in the serum were also associated with a reduced risk of ARM. The investigators hypothesized that the stronger association of plasma zeaxanthin (when compared with lutein) with ARM may be related to the fact that the central retina preferentially accumulates zeaxanthin, and that zeaxanthin appears to be a better photoprotector during prolonged exposure to short wavelength visible light. The finding that serum zeaxanthin may be more important than serum lutein in any protection conferred against ARM is also consistent with the recent report by Gale et al. [174]. In the cross-sectional arm of the Rotterdam Eye Study, no association between ARM and serum levels of either beta-carotene or lutein or zeaxanthin was observed [235].

In summary, the relationship between ARM and dietary intake (and serum levels) of macular carotenoids remains unclear. However, recent reports from AREDS and the Blue Mountains Eye Study do suggest that dietary intake of lutein and zeaxanthin may be protective for ARM.

**(e) Antioxidant Enzymes**

Several antioxidant enzymes form vital components of cellular defense mechanisms against oxidative stress [173]. There is scant evidence from population-based epidemiological studies for any association between these enzymes and ARM. The POLA study found that high levels of plasma glutathione peroxidase were significantly associated with a nine-fold decrease in the prevalence of late ARM [29]. However, that study failed to find any association between high levels of superoxide dismutase and the prevalence of either early or late ARM [29].

**(f) Zinc (Table 13)**

The final aspect of antioxidant status that has been evaluated in population-based epidemiologic studies with respect to ARM is that of the trace element, zinc is an essential cofactor for antioxidant enzymes. Food rich in zinc includes meat, poultry, fish, whole grains, and dairy products [173].

The retrospective arm of the Beaver Dam Eye Study detected a weak protective effect for individuals in the highest quintile of dietary intake of zinc when compared with those in the lowest quintile (Table 13) [234]. However, this was confirmed only for pigmentary macular changes in the prospective arm of that study [234]. Of note, the Blue Mountains Eye Study found no association between dietary intake of zinc and either the prevalence or incidence of ARM [226]. In contrast, the Rotterdam Study reported that a high dietary intake of zinc had a protective effect for the incidence of ARM [235].

#### 5.2.2. Pro-Oxidant Status

Aspects of pro-oxidant status that have been evaluated with respect to risk for ARM in population-based epidemiologic studies include exposure to light and dietary intake of fatty acids.

**(a) Dietary Intake of Fatty Acids (Table 14)**

Polyunsaturated fatty acids are particularly susceptible to free radical damage, and it is noteworthy that the photoreceptor membranes of both rods and cones contain a lipid bilayer [224]. Polyunsaturated fatty acids account for about 50% of the lipid bilayer of rod outer segment membranes, and the concentrations of these molecules within this layer can be altered by dietary modification [224]. Therefore, dietary intake of fatty acids may be associated with ARM by enhancing the vulnerability of rod outer segment membranes to oxidative damage [254, 256]. However, animal studies have shown that certain polyunsaturated fats, particularly docosahexaenoic acid, may actually confer resistance against retinal oxidation, probably because they replenish these compounds in the photoreceptor membranes following their depletion by free radical injury [224]. Docosahexaenoic acid is found predominantly in oily fish and offal [224]. Dietary intake of saturated fats could be etiologically important for the development of ARM because of the effect of such intake on cholesterol levels within the bloodstream or within the tissue, or through effects on the inflammatory cascade.

A number of studies have investigated dietary intake of total fat, saturated fat, and polyunsaturated fat with respect to ARM (Table 14). Further, some of these studies have also specifically investigated dietary intake of fish and fish oils with respect to risk for ARM. Cross-sectional evidence from the Beaver Dam population suggested that high intake of saturated fats and cholesterol was associated with increased prevalence of early ARM [254]. In NHANES III, similar relationships between total fat intake and early ARM were seen, but these did not reach statistical significance [56]. In the Blue Mountains population, a significant, but marginal, relationship between total dietary intake of fat and early ARM was initially detected [253, 255]. More recently, data from the Blue Mountains Eye Study failed to detect an association between high dietary intake of total fat, or subtypes of fat, and the incidence of ARM (early or late) [253]. However, the Blue Mountains Eye Study did reveal a protective, albeit weak, effect of dietary intake of fish for the incidence of late ARM [253, 255].

**(b) Light (Table 15)**

The retina is subjected to high levels of cumulative irradiation with visible light over a lifetime. Exposure to ambient light is known to result in retinal injury, and a number of laboratory studies indicate that oxidative stress plays a role in this process [173]. Furthermore, the vulnerability of the retina to damage by visible light varies with the wavelength of the incident light, and it has been shown that the power required to cause photo-oxidative retinal damage is up to 1000 times lower for blue-light than for infrared wavelengths, depending on the duration of exposure [257–259]. On this basis, it has been postulated that cumulative exposure to visible light, especially (short-wavelength) blue-light, is etiologically important for the pathogenesis of ARM, and this hypothesis has been explored in a number of epidemiologic studies (Table 15).

Unfortunately, it is difficult to accurately measure cumulative and lifetime exposure to visible light. The Beaver Dam Eye Study investigated how exposure to sunlight relates to prevalence and incidence (5 and 10 years) of ARM and found significant associations between extended exposure to summertime sun and the 10-year incidence of early ARM [258, 260, 261]. The investigators did not, however, detect any relationship between mean annual ambient UV-B exposure and the incidence or progression of ARM at 10 years [258, 260, 261]. In contrast to the findings in the Beaver Dam Eye Study, the POLA study [30] found that sunlight exposure was not significantly associated with increased risk of ARM [262]. In fact, in the POLA Study, the risk of early ARM [262] was lower in subjects exposed to high ambient solar radiation. In the Visual Impairment Project, people with ARM were found to have had higher annual ocular exposure to sunlight, but this finding was not statistically significant [32].

## 6. Ocular Factors

### 6.1. Refractive Error (Table 16)

It has been proposed that refractive error may be etiologically important for the development of ARM [267].

#### 6.1.1. Hypothesis/Rationale

The pathophysiologic mechanisms for any association between refractive error and ARM remain unclear. Hypermetropic eyes typically have a smaller axial length than emmetropic or myopic eyes, and have a tendency towards increased scleral thickness [267, 268]. These factors may contribute to an increased scleral rigidity in hypermetropic eyes, which may, in turn, be associated with an increase in the resistance of choroidal venous outflow and therefore be consistent with the vascular hypothesis of ARM pathogenesis [267, 268].

#### 6.1.2. Epidemiologic Studies

A number of case-control studies have looked at the association between hypermetropia and ARM [121]. However, the results have been inconsistent. A small number of population-based studies have also examined the relationship between hypermetropia and ARM (Table 16). In these studies, autorefractor readings and subjective refraction were used to assess spherical equivalent refractive error (in diopters). In 1998, the Blue Mountains Eye Study showed a weak association between hypermetropia and early ARM only [263]. However, five-year followup of the same study failed to show any association between hypermetropia and the incidence of either late or early ARM [265]. 

Similarly, the Beaver Dam Eye Study failed to identify a relationship between refractive status and either the 5-or 10-year incidence of ARM (early or late) [266]. In contrast to this, the Rotterdam study demonstrated that hypermetropia was associated with an increased prevalence of ARM, and, to a slightly lesser extent, with an increased incidence of ARM [264]. Indeed, this study showed that, for each diopter increase in hypermetropia, there was a 5% increase in the risk of developing incident ARM. Finally, cross-sectional data from the Beijing Eye Study revealed that (other than age) hypermetropic refractive error was the single most important risk factor for ARM [37].

### 6.2. Iris Color (Table 17)

Iris color is related to skin pigmentation, and therefore a relationship, if any, between iris color and ARM must be interpreted with full appreciation of the possibility that any observed association between iris color and ARM may actually reflect ethnicity-based differences for this condition.

#### 6.2.1. Hypothesis/Rationale

Subjects with nonblue irides have increased tissue concentrations of melanin, and this is also evident in the choroid [275, 276]. It has been hypothesized that choroidal melanin may protect against oxidative damage from sunlight, thus providing protection to the retina and reducing the risk of ARM [275, 276]. Heavy iris pigmentation has also been shown to reduce the amount of light entering the eye [275, 276], and this is another possible protective mechanism. Also, some papers have reported that light iris color is associated with a relative lack of macular pigment, and some investigators have speculated that this relative lack of antioxidant defenses in the central retina may also explain any increased risk for ARM in association with light iris color [275, 276].

#### 6.2.2. Epidemiologic Studies

Initial case-control studies suggested that brown-colored irides were associated with a reduced risk for ARM [276]. In 1990, however, the first population-based study investigating this found no significant difference in the prevalence of ARM between participants with brown- or blue-colored irides (Table 17) [269]. Indeed, initial evidence from the Beaver Dam Eye Study also suggested the absence of any significant association between iris color and risk for ARM [21]. However, in 1998, cross-sectional data from the Blue Mountains Eye Study reported that blue iris color was significantly associated with an increased risk of both early and late ARM when compared with dark irides [270, 275]. Then, a second and prospective paper from the Beaver Dam Eye Study, published in 2003, demonstrated that people with brown irides were significantly more likely to develop soft indistinct drusen, but less likely to develop retinal pigment epithelial changes, than those with light irides at five-year followup [271, 275]. However, this paper did not show any association between iris color and the development of late ARM [271, 275].

There is a strong correlation between hair color and iris color, thus prompting a number of researchers to investigate hair color with respect to risk for ARM. The Beaver Dam Eye Study showed that people with brown hair were less likely to develop early ARM, in the form of pigmentary abnormalities, when compared to those with blond hair [271, 275]. In that study, however, hair color was not associated with the development of late ARM [271, 275].

### 6.3. Cataract and Cataract Surgery (Tables 18 and 19)

Cataract and ARM are the most frequent causes of decreased vision in older people. Of the numerous cataract subtypes, the most commonly seen are nuclear sclerosis, cortical lens opacities, and posterior subcapsular cataracts. The prevalence of lens opacification rises with increasing age and may therefore have risk factors common with ARM. Alternatively, cataracts could, in theory at least, have a protective effect against ARM due to their light-absorbing properties.

#### 6.3.1. Hypothesis/Rationale

It has been hypothesized that cataracts may provide protection against ARM by absorbing blue light, and thus reducing oxidative damage to the retina [281–284]. However, this protective effect, if any, would be restricted to certain types of cataract, such as nuclear sclerosis. 

As sunlight has been putatively implicated in the pathogenesis of ARM, the possibility that cataract surgery results in increased risk of ARM has been postulated. Indeed, ophthalmologists often observe the development of neovascular AMD shortly following cataract surgery. Such a hypothesis has prompted lens manufacturers to incorporate a blue-light filter into the intraocular lens. However, the benefit of such blue-filtering intraocular lenses has yet to be established [281, 282]. Cataract surgery is also associated with intraocular inflammatory changes, which may be associated with progression of ARM. Alternatively, any association between ARM and cataract surgery may simply be due to the fact that identification of ARM may have gone unnoticed preoperatively, because of impaired fundus visualization attributable to the lens opacity.

#### 6.3.2. Epidemiologic Studies

Early population-based epidemiological studies have reported inconsistent findings on the relationship between cataract and ARM (Table 18). Cross-sectional data from the Framingham Eye Study reported that nuclear sclerosis was negatively associated with ARM, but that cortical lens opacities were positively associated with this condition [52, 53]. In the National Health and Nutrition Examination Survey I, subjects with either form of cataract were at increased risk for development of ARM [54]. The inconsistencies reported earlier may be due, in part, to the lack of any objective means of recording or grading the lens opacities in these studies.

However, these limitations have been addressed in more recent studies. In 1994, the Beaver Dam Eye Study published evidence indicating that nuclear sclerosis was associated with an increased prevalence of early ARM, but not of late ARM [273, 274]. The investigators did not, however, find any relationship between either cortical or posterior subcapsular cataracts and ARM (early or late). In 2002, the investigators went on to show that nuclear sclerosis at baseline was associated with an increased 10-year incidence of early ARM, but no association with the incidence of late ARM was reported [20, 273, 274]. In 2005, the Copenhagen City Eye Study demonstrated a positive association between baseline cataract and the 14-year incidence of ARM [38–40]. In contrast to the earlier studies, however, the Blue Mountains Eye Study found no association between the presence of cortical, nuclear, or posterior subcapsular cataract and ARM (either early or late) [272]. 

Evidence for a relationship between cataract surgery and ARM is, however, more consistent (Table 19). A single large population-based study reported no association between cataract surgery and ARM, but most other studies have shown some association [278, 279]. Pooled data from the Salisbury Eye Evaluation, the Proyecto VER, and the Baltimore Eye Survey showed that cataract surgery at baseline examination was associated with an increased prevalence of late ARM [277]. Evidence from the population-based Andhra Pradesh Eye Disease Study also demonstrated an association between prior cataract surgery and prevalence of ARM [41]. Indeed, longitudinal data from the Beaver Dam Eye Study has also demonstrated a relationship between prior cataract surgery and the incidence of late ARM at both 5- and 10-year followup [278]. However, this latter study did not find a relationship between prior cataract surgery and incidence of early ARM. When 5-year incidence data from the Beaver Dam Eye Study was pooled with similar data from the Blue Mountains Eye Study, pseudophakic and aphakic eyes were at nearly six-fold increased risk for developing late ARM when compared with phakic eyes [277]. Furthermore, 10-year followup in the Blue Mountains Eye study revealed that pseudophakic eyes had a three-fold increased risk of developing late ARM when compared with phakic eyes [280]. However, it should be noted that a recent report from AREDS data failed to detect an association between cataract surgery and ARM [121, 280]. 

In conclusion, the relationship between cataract and cataract surgery and AMD has been the subject of much debate over recent years. With regard to cataract and ARM, some studies show cataract to be negatively associated with ARM, others having equivocal results. With regard to cataract surgery and ARM, some studies show it to be beneficial in AMD patients, whereas others show cataract extraction to have deleterious effects and result in progression of disease. At this time however, it is not possible to draw reliable conclusions from the available data to determine whether cataract surgery is beneficial or harmful in people with AMD. Physicians will have to make practice decisions based on best clinical judgment until appropriate studies are conducted and reported.

## 7. Miscellaneous Factors

### 7.1. Alcohol Consumption (Table 20)

The association, if any, between alcohol consumption and ARM has been investigated in a number of studies.

#### 7.1.1. Hypothesis/Rationale

Alcohol has been shown to have both harmful and beneficial effects with respect to a number of conditions, and may exert deleterious effects by interfering with cellular defense mechanisms against oxidative processes [289, 290].

However, investigation of the association between alcohol and cardiovascular disease has also revealed a number of mechanisms whereby alcohol may actually exert beneficial effects. Putative mechanisms leading to a protective effect of alcohol include increased HDL cholesterol, decreased platelet aggregation [289, 290], and decreased levels of fibrinogen. Indeed, different forms of alcoholic beverage may exert different influences. Wine has high concentrations of phenolic compounds, and these are thought to have beneficial effects. Beer, on the other hand, contains nitrosamines, which may exert toxic effects. However, when investigating the relationship between alcohol and ARM, it should be borne in mind that alcohol consumption is also an indicator of other lifestyle and dietary variables.

#### 7.1.2. Epidemiologic Studies

Initial evidence from the Beaver Dam Eye Study revealed that consumption of beer in the past year was associated with an increased prevalence of pigmentary changes at the macula, and of neovascular AMD (Table 20) [285]. However, the investigators did not find any association between current consumption of wine or liquor with early or late ARM. The 5-year incidence data from this study subsequently showed that, with the exception of an association between beer drinking and retinal drusen in men only, consumption of alcoholic beverages was unrelated to risk for ARM [288]. However, 10-year data from the same study detected an association between heavy alcohol consumption and incident late ARM [116]. The Blue Mountains Eye Study found a significant and positive association between consumption of liquor and early ARM, but no link between beer consumption and early ARM [286]. NHANES I reported that moderate wine consumption was associated with a protective effect against the development of ARM (unspecified whether early or late) [287]. 

Other studies have also investigated the effects of total alcohol consumption with respect to age related maculopathy. Cross-sectional data from the Reykjavik Eye Study found that current alcohol consumption was associated with decreased risk of drusen formation, and increased risk of pigmentary changes, at the macula [34, 35]. The Copenhagen City Eye Study found that excessive alcohol consumption increased the risk of early ARM [38, 39]. Similarly, cross-sectional data from Los Angeles Latino Eye Study found that heavy drinking was associated with increased risk of retinal pigment epithelial depigmentation and with a higher prevalence of late ARM (atrophic or neovascular) [114]. The Andhra Pradesh Eye Study found that the prevalence of ARM was significantly lower in light-alcohol drinkers when compared with nonalcohol drinkers [41].

### 7.2. Medication Use

The association between use of certain medications and risk of ARM has been investigated in a number of population-based epidemiologic studies [23].

#### 7.2.1. Hypothesis/Rationale

Certain medications, such as chloroquine and chlorpromazine, are well known to have toxic effects on the human retina [291]. It is biologically plausible, therefore, that the use of medications may be relevant to the risk of developing retinal disease, such as ARM. However, study of the association between medication use and ARM may also reflect an association between ARM and the disease for which the medication has been prescribed.

#### 7.2.2. Epidemiologic Studies

A review of studies investigating the relationship between ARM and hormone replacement therapy and lipid-lowering agents has already been discussed.

The Beaver Dam Eye Study investigated the association, if any, between medication use and the 5-year incidence of early ARM [168]. There was no detectable relationship between early ARM and intake of the following medications: most antihypertensive drugs, most central nervous system medications, nonsteroidal anti-inflammatory medications, estrogens. and lipid lowering agents. However, the investigators did report a protective effect of antidepressants with respect to the incidence of early ARM [168]. The Blue Mountains Eye Study failed to detect an association between the use of systemic anti-inflammatory medications and the prevalence or incidence of ARM [196]. In the Visual Impairment Project, past or current use of angiotensin-converting enzyme inhibitors was associated with increased risk of both early and late ARM [32]. However, these findings were not adjusted for blood pressure readings, as this information was not available for that study.

Due to the difficulties and limitations in the studies designed to investigate associations between medication usage and ARM, data from the Beaver Dam Eye Study, the Blue Mountains Eye Study, and the Rotterdam Study has been pooled [26]. This pooled data failed to reveal a strong association between usage of any medicines and the incidence of early ARM [26]. However, pooling of the data revealed a slightly increased risk of early ARM among users of *β*-blockers, and a reduced risk of early ARM amongst users of hormone replacement therapy and tricyclic antidepressants. However, it should be emphasized that these latter findings were of borderline significance.

### 7.3. Coffee Consumption

Intake of caffeine has been shown to have a vasoconstrictive effect on the retinal capillary circulation at the macula [292], thus prompting the notion that cumulative intake of coffee is associated with risk for ARM (in view of the vascular hypothesis for this condition). The Beaver Dam Eye Study investigated coffee consumption with respect to risk for ARM [293], and it was reported that coffee and caffeine consumption were not associated with the 5-year incidence of early ARM.

### 7.4. Frailty

ARM can also be considered to be part of a global aging process, as well as a specific disease entity. In this context, markers of frailty or biologic aging may be relevant to risk for ARM. Such markers include gait-time, handgrip strength, peak expiratory flow rate, and the ability to stand from a sitting position without using the arms. The Beaver Dam Eye Study has examined the relationship between these markers and ARM [223], and the investigators reported a weak but significant association between handgrip strength and risk for ARM in men (after adjusting for comorbidity). The investigators proposed therefore that ARM should probably be viewed as a specific disease process (albeit age-related).

## 8. Conclusion

Meaningful comment on risk for ARM has been greatly facilitated by a large number of studies of varying design that have been published over the last twenty years. Our review of case series, large-scale population-based studies, cohort, cross-sectional, and case-control studies strongly indicate that age, tobacco use, and family history of ARM represent indeed the risk for this condition. Further, there is a growing body of evidence that cataract surgery and obesity also represent risk for ARM, especially the neovascular form of this disease. 

However, population-based epidemiologic studies have failed to consistently demonstrate associations between ARM and a myriad of other potential risk factors. It should be noted that the low prevalence of late ARM in the general population makes the detection of relationships with potential risk factors for its development particularly difficult in the context of population-based studies. Moreover, identification of relationships may also be confounded where disease expression is strongly determined by genetic background. Indeed, our recently enhanced understanding of the genetic basis of ARM will inform the design and interpretation of future studies attempting to investigate risk for ARM, and the role of gene-environment interaction in the etiopathogenesis of this condition.

##  Method of Literature Search

References for this review were identified through a comprehensive systematic literature search of the electronic MEDLINE database (1966–2008) using the Pubmed search service. Further articles, abstracts, and textbook references generated from reviewing the bibliographies of the initial search were selectively included. To ensure the up-to-date nature of our review article, current issues of *Archives of Ophthalmology, Survey of Ophthalmology, American Journal of Ophthalmology, Ophthalmology, British Journal of Ophthalmology, and Investigative Ophthalmology* and *Visual Sciences* were regularly reviewed throughout the period of writing. The following key words, and combinations thereof, were used to perform the initial search: *age-related maculopathy, agerelated macular degeneration, risk factors, epidemiology, family history, smoking, cardiovascular disease, inflammation, oxidative stress, cataract, iris color, refractive error, and alcohol.*

## Figures and Tables

**Table 1:** Large epidemiologic studies investigating risk for ARM.

Study	Location	Design	No. of participants
Beaver Dam Eye Study [17–22]	USA (1988–2005)	Cross-Sectional	4926

		Prospective	3684 (5-year)
			2764 (10-year)
			2119 (15-year)

Blue Mountains Eye Study [23–25]	Australia (1992–2004)	Cross-Sectional	3654
		Prospective	2335 (5-year)
			1952 (10-year)

Rotterdam Study [23, 26–28]	Holland (1990–2004)	Cross-Sectional	6418
		Prospective	4953 (2-year)
			3406 (6.5-year)
			2387 (11-year)

Pathologies Oculaires Liees a L'Age [29, 30]	France (1995–2000)	Cross-Sectional	2584
		Prospective	1642 (3-year)

Los Angeles Latino Eye Study [31]	USA (2000–2003)	Cross-Sectional	6357

Melbourne Visual Impairment Project [32, 33]	Australia (1992-1999)	Cross-Sectional	5147
		Prospective	3271 (5-year)

Reykjavik Eye Study [34–36]	Iceland (1996–2001)	Cross-Sectional	1045
		Prospective	846 (5-year)

Beijing Eye Study [37]	China (2001)	Cross-Sectional	4439

Copenhagen City Eye Study [38–40]	Denmark (1986–2002)	Cross-Sectional	946
		Prospective	359 (14-year)

Andhra Pradesh Eye Disease Study [41]	India (1996–2000)	Cross-Sectional	3723

Barbados Eye Studies [42–45]	Barbados (1987–2003)	Cross-Sectional	4631
		Prospective	3427 (4-year)
			2793 (9-year)

Salisbury Eye Evaluation Project [46]	USA (1993)	Cross-Sectional	2520

Proyecto VER [47]	USA (1997–1999)	Cross-Sectional	4774

Baltimore Eye Survey [5]	USA (1985–1988)	Cross-Sectional	5308

Aravind Comprehensive Eye Survey [48]	India (1995–1997)	Cross-Sectional	5150

European Eye Study [1, 49]	7 European Countries (2000–2003)	Cross-Sectional	5040

Hisayama Study [50, 51]	Japan (1998–2003)	Cross-Sectional	1482
		Prospective	961 (5-year)

Framingham Eye Study [52, 53]	USA (1973–1975)	Cross-Sectional	2940

National Health and Nutrition Examination Survey I [54]	USA (1971–1972)	Cross-Sectional	3056

National Health and Nutrition Examination Survey III [55, 56]	USA (1988–1994)	Cross-Sectional	8270

Cardiovascular Health Study [57, 58]	USA (1997–1998)	Cross-Sectional	2361

Atherosclerosis Risk in Communities Study [19, 59, 60]	USA (1993–1995)	Cross-Sectional	11532

MRC Trial of Assessment and Management of Older People in the Community [2]	UK (1996–2000)	Cross-Sectional	13900

**Table 2:** Risk factors for ARM examined in the cited studies.

*Genetic predisposition*

Family history of ARM
Complement Factor H gene
Apolipoprotein E gene
LOC gene

*Cardiovascular disease*

Clinical Evidence of Atherosclerosis
Angina/Heart attack/Stroke
Subclinical evidence of atherosclerosis
Carotid atherosclerosis
Aortic atherosclerosis
Cigarette smoking
Diabetes mellitus
Hypertension and associated disease
Ischemic cerebral white matter changes
Abnormalities of the retinal vasculature
Cholesterol
Total cholesterol
Low-density Lipoprotein (LDL) cholesterol
High-density Lipoprotein (HDL) cholesterol
Obesity
Female sex hormones
Endogenous estrogen exposure
Age at menarche
Age at menopause
Number of pregnancies
Exogenous estrogen exposure
Oral contraceptives
Hormone replacement therapy
Novel risk factors for atherosclerosis
Lipid-related Factors
Apolipoproteins
Lipoproteins
Inflammatory markers
C-reactive protein
Interleukins
Serum Amyloid A
Vascular and Cellular Adhesion Molecules
White Blood Cell Count
Homocysteine/Folate/Vitamin B12/Vitamin B6
Infectious agents
Cytomegalovirus
Helicobacter pylori
Chlamydia pneumoniae

*Indicators of Inflammation*

Systemic Diseases with Inflammatory Components
Gout
Emphysema
Anti-inflammatory Medications
NSAIDs
Steroids
Markers of Systemic Inflammation
White Blood Cell Count
Serum Albumin
Plasma Fibrinogen
C-reactive Protein
Complement Factor H Y402H Polymorphism
CRP Haplotype
Serum Amyloid A
Interleukin-6
Tumor Necrosis Factor-*α*

*Markers of Endothelial Dysfunction*

Intercellular Adhesion Molecule-1
E-Selectin

*Indicators of Oxidative Stress*

Anti-oxidants
Vitamin C
Vitamin E
Vitamin A
Carotenoids
Lutein
Zeaxanthin
*α*- and *β*-Carotene
*β*-Cryptoxanthin
Lycopene
Enzymes
Plasma Glutathione Peroxidase
Superoxide Dismutase
Trace Elements
Zinc
Pro-oxidant status
Dietary fat intake
Total fat
Saturated fat
Polyunsaturated fat
Fish/Fish oils
Visible Light Exposure
Sunlight
Ultraviolet-B

*Ocular Factors*

Refractive Error
Emmetropia
Myopia
Hypermetropia
Iris Color
Cataract
Nuclear Sclerosis
Cortical Lens Opacities
Posterior Subcapsular Cataracts
Cataract Surgery

*Miscellaneous Factors*

Alcohol Consumption
Beer
Wine
Spirits
Medication Use
Estrogens
Lipid-lowering Agents
CNS Medications
Non-steroidal Anti-Inflammatory Medications
Anti-hypertensive Medications
Coffee Consumption
Frailty
Physical Activity

**Table 3:** Studies investigating the relationship between tobacco use and risk for ARM.

Study	No. of cases		Outcome measure/risk factor	Type of ARM	Odds ratio or relative risk	95% confidence interval
Cross-Sectional studies						

Beaver Dam Eye Study [110]	4771		Current smokers versus ex-smokers or never smokers	Neovascular AMD	2.50	1.01–6.20 (Females)
					3.29	1.03–10.50 (Males)

Blue Mountains Eye Study [24, 111]	3654		Current smoker versus current nonsmoker	Late ARM	3.92	2.07–7.41
				Early ARM	1.75	1.20–2.54

Pathologies Oculaires Liees a L'Age [112]	2196		Current smokers	Late ARM	3.6	1.1–12.4

Melbourne Visual Impairment Project [26]	5147		Smoked Cigarettes for longer than 40 years	Late ARM	2.39	1.02–5.57

Rotterdam Study [113]	6174		Current smokers	Neovascular AMD	6.6	2.8–15.9

Los Angeles Latino Eye Study [114]	5875		Ever smoked	Late ARM	2.4	1.03–5.4

			Ever smoked versus never smoked	All AMD	2.4	*P* < .5
Copenhagen Study Denmark [39, 40]	773			Atrophic AMD	2.5	*P* < .5
				Neovascular AMD	1.5	NS

Chesapeake Bay, Waterman Study, USA [46]	769		Ever smokers versus never smokers	All AMD	0.61	(0.35–1.05)

Prospective studies						

Blue Mountains Eye Study [111, 115]	2335		Current smoker versus never smoker	Geographic atrophy	3.6	1.1–11.3
				Any late ARM	2.5	1.0–6.2

Beaver Dam Eye Study [22, 116]	2764		Current smoker	Late ARM	0.51	0.18–1.46
				Early ARM	1.37	0.98–1.94

*Pooled data*: Beaver Dam Eye Study/Blue Mountains Eye Study/Rotterdam Study [23, 26]	14752		Current Smoker	Late ARM	3.12	2.10–4.64

*Pooled data*: Beaver Dam Eye Study/Blue Mountains Eye Study/Rotterdam Study [23, 26]	9523		Current smoker	Late ARM	2.35	1.30–4.27

Physician's Health Study, USA [117]			Current smokers < 20/d versus never smokers	All AMD	1.26	0.61–2.9
	21157		Current smokers > 20/d versus never smokers	All AMD	2.46	1.6–3.79
				Neovascular AMD	1.95	0.89–4.2

Nurse's Health Study, USA [118]	31843		Currrent smokers versus never smoked	All AMD	1.7	1.2–2.50

Hisayama Study [50]	961		Current or ex-smoker	ARM	2.2	1.14–4.33
Case-Control Studies	Case	Con	Current smokers versus never smokers			

France [112]	26	23		Neovascular AMD	1.25	0.3–4.4

Eye Disease Case-Control Study [119]	421	615		Neovascular AMD	2.2	1.4–3.5

France [112]	1844	1844		All ARM/AMD	1.09	0.83–1.42
				Neovascular AMD	2.97	1.0–8.84

Japan [120]	56	82		Early ARM	1.25	1.09–1.44
				Atrophic AMD	1.61	1.06–2.42

Age-related Eye Disease Study [121]	340	111		Neovascular AMD	1.91	1.57–2.33
	5					

**Table 4:** Studies investigating the relationship between hypertension and risk for ARM.

Study	No. of cases	Measure of risk factor	Type of ARM	Odds ratio or relative risk	95% confidence interval
Cross-Sectional Studies					

Hisayama Study [51]	1482	Hypertension (history or examination)	ARM	1.58	1.03–2.41 (Males)

Pathologies Oculaires Liees a L'Age [122]	2584	Systolic BP (per 10 mm of Hg)	Late ARM	1.19	0.98–1.43
		Diastolic BP (per 10 mm of Hg)	Late ARM	0.98	0.72–1.35

Blue Mountains Eye Study [24, 26]	3654	Previous diagnosis or systolic BP > 160 mm Hg or diastolic BP > 90 mm Hg	Early ARM	0.88	0.67–1.16
			Late ARM	1.06	0.63–1.79

Beaver Dam Eye Study [110]	4926	Hypertension	Neovascular AMD	0.79	0.44–1.42
			Geographic atrophy	1.07	0.46–2.47

Prospective Studies					

*Pooled data*: Beaver Dam Eye Study/Blue Mountains Eye Study/Rotterdam Study [23, 26]	9523	Systolic blood pressure (per 2 mm of Hg)	Late ARM	1.03	0.94–1.13
		Diastolic blood pressure (per 10 mm of Hg)	Late ARM	0.95	0.79–1.16

Rotterdam Study [23, 123, 124]	4822	Elevated systolic blood pressure (per 10 mm Hg increase)	ARM	1.06	1.01–1.12
		Pulse pressure (per 10 mm Hg increase)	ARM	1.09	1.02–1.15

Beaver Dam Eye Study [125, 126]	2764	High systolic blood pressure at baseline	Retinal pigment epithelial depigmentation	1.10	1.01–1.18
			Neovascular AMD	1.22	1.06–1.41

**Table 5:** Studies investigating the relationship between cholesterol and risk for ARM.

Study	No. of cases	Measure of risk factor	Type of ARM	Odds ratio or relative risk	95% confidence interval
Cross-Sectional Studies					

Cardiovascular Health Study [58]	2361	Serum total cholesterol (per 10 mg/dL increase)	ARM	0.95	0.91–0.98

National Health and Nutrition Examination Survey III [54]	8270	HDL cholesterol (per mmol/L)	Early ARM	1.30	0.99–1.71
		Triglycerides (per mmol/L)	Soft drusen	0.88	0.79–0.99

Pathologies Oculaires Liees a L'Age [122]	2584	Cholesterol (per mmol/L)	Soft drusen	1.07	0.97–1.17
			Late ARM	0.97	0.71–1.31
		HDL Cholesterol (per mmol/L)			

Blue Mountains Eye Study [26]	3654	Cholesterol (per 10 mg/dL)	Early ARM	0.95	0.84–1.09
			Late ARM	1.08	0.92–1.27

Prospective studies					

Beaver Dam Eye Study [160]	3684	Serum Cholesterol (per 10 mg/dL increase)	Geographic Atrophy	1.29	1.05–1.58

Rotterdam Eye Study [161, 162]	4776	Serum Cholesterol (per 10 mg/dL)	Early ARM	0.96	0.84–1.09
		HDL Cholesterol (per mmol/L)	Early ARM	1.38	0.92–1.79

**Table 6:** Studies investigating the relationship between obesity and risk for ARM.

Study	No. of cases	Measure of risk factor	Type of ARM	Odds ratio or relative risk	95% confidence interval
Cross-Sectional Studies					

Beaver Dam Eye Study [128]	4926	BMI	Neovascular AMD	1.02	0.97–1.08
			Geographic atrophy	1.06	0.98–1.14

Blue Mountains Eye Study [26]	3654	BMI > 30	Early ARM	1.78	1.19–2.68
		BMI < 20	Early ARM	1.92	1.16–3.18

Pathologies Oculaires Liees a L'Age [163]	2584	BMI > 30	Late ARM	2.29	1.00–5.23
			Retinal pigmentary abnormalities	1.54	1.05–2.26

Prospective Studies					

Beaver Dam Eye Study [18, 20, 125]	3583	BMI	Retinal Pigmentary Abnormalities	1.03	1.00–1.06

*Pooled data*: Beaver Dam Eye Study/Blue Mountains Eye Study/Rotterdam Study [26]	9523	BMI > 30	Late ARM	0.87	0.51–1.47

**Table 7:** Studies investigating the relationship between female sex hormones and risk for ARM.

Study	No. of cases	Measure of risk factor	Type of ARM	Odds ratio or relative risk	95% confidence interval
Cross-Sectional Studies					

Beaver Dam Eye Study [180]	4926	Number of pregnancies	Early ARM	0.95	0.92–1.01
		Hormone replacement therapy usage	Late ARM	0.94	0.63–1.39

Blue Mountains Eye Study [24, 188]	3654	Years from menarche to menopause	Early ARM	0.97	0.95–0.99

Los Angeles Latino Eye Study [114]	5875	Use of oral contraceptive pills	Early ARM	0.5	0.4–0.8
		Use of hormone replacement therapy	Early ARM	0.8	0.6–1.2

Pathologies Oculaires Liees a L'Age [189]	2584	Hormone replacement therapy	Late ARM	N/A	N/A

Salisbury Eye Evaluation Project [190]	1458	Current use of hormone replacement therapy	Early ARM	0,7	0.3–1.5
			Late ARM	0.6	0.1–2.9

Aravind Comprehensive Eye Survey [191]	5539	Age at menarche 14+	ARM	2.3	1.2–4.7
		Age at menopause <45 years	ARM	1.5	0.3–8.1
		Endogenous estrogen exposure <30 years	ARM	2.2	0.4–12.0

Prospective Studies					

Beaver Dam Eye Study [181]	3684	Hormone replacement therapy (3+ years)	Early ARM	0.98	0.56–1.73
			Late ARM	1.30	0.36–5.21

Rotterdam Study [23, 26, 192]	4616	Early menopause following oophorectomy	ARM	3.8	1.1–12.6

*Pooled data*: Beaver Dam Eye Study/Blue Mountains Eye Study/Rotterdam Study [23, 26]	9523	Time from menarche to menopause per year	Late ARM	0.99	0.95–1.03
		Hormone replacement therapy	Late ARM	1.00	0.40–2.45

**Table 8:** Studies investigating the relationship between indicators of inflammation and risk for ARM.

Study	No. of cases	Measure of risk factor	Type of ARM	Odds ratio or relative risk	95% confidence interval
Cross-Sectional Studies					

Atherosclerosis Risk in Communities Study	11264	White blood cell count	Early and late ARM	N/A	N/A

National Health and Nutrition Examination Survey III [26, 54]	8270	Serum albumin	Early ARM	0.97	0.95–1.00

Cardiovascular Health Study [193]	2361	Serum albumin	Early ARM	0.59	0.39–0.92
		Plasma Fibrinogen	Early ARM	N/A	N/A

Cardiovascular Health Study [193]	2755	C-reactive protein	Early and Late ARM	1.24	0.87–1.78 (4th Quartile)

Beaver Dam Eye Study [194, 195]	2764	Gout	Early ARM	0.69	0.41–1.16
			Late ARM	2.18	1.06–4.47
		Emphysema	Early ARM	1.11	0.49–2.51
			Late ARM	2.55	0.74–8.81

Beaver Dam Eye Study [194, 195]	3684	NSAIDs	Early ARM	1.04	0.78–1.38
		Aspirin	Early ARM	1.08	0.79–1.48
		Oral steroids	Early ARM	0.80	0.28–2.28

Blue Mountains Eye Study [196]	2313	Steroids or NSAIDs	Early ARM	1.2	0.8–1.6
			Late ARM	0.9	0.4–1.9

Beaver Dam Eye Study [194, 195]	2764	White blood cell count	Early ARM	1.03	0.98–1.10
			Late ARM	0.99	0.86–1.14
		Serum albumin	Early ARM	0.91	0.64–1.29
			Late ARM	0.52	0.24–1.14

**Table 9:** Studies investigating the relationship between vitamin C and risk for ARM.

Study	No. of cases	Measure of risk factor	Type of ARM	Odds ratio or relative risk	95% confidence interval
Cross-Sectional Studies					

National Health and Nutrition Examination Survey I [54]	3082	Dietary intake	Early and late ARM	1.15	0.79–1.66

Blue Mountains Eye Study [233]	2900	Dietary Intake	Early ARM	0.9	0.5–1.4
			Late ARM	1.3	0.5–3.4

Prospective Studies					

Beaver Dam Eye Study [234]	1968	Dietary intake	Early ARM	0.8	0.5–1.2
			Late ARM	0.6	0.2–2.0

Blue Mountains Eye Study [226, 233]	1989	Dietary intake	Early ARM	2.3	1.3–4.0

Rotterdam Study [235]	4170	Dietary intake of vitamin C in combination with vitamin E, *β*-carotene and zinc	Early and late ARM	0.65 (Hazard Ratio)	0.46–0.92

Baltimore Longitudinal Study of Aging [231]	678	Fasting plasma level	Early and late ARM	0.55	0.28–1.08

Pathologies Oculaires Liees a L'Age [29, 236]	1791	Fasting plasma level	Late ARM	0.89	0.32–2.5

**Table 10:** Studies investigating the relationship between vitamin E and risk for ARM.

Study	No. of cases	Measure of risk factor	Type of ARM	Odds ratio or relative risk	95% confidence interval
Cross-Sectional Studies					

Pathologies Oculaires Liees A L'Age [29, 236]	2157	Lipid-standardized plasma level	Late ARM	0.18	0.05–0.07

Prospective Studies					

Beaver Dam Eye Study [237]	1968	Dietary intake		0.7	0.5–1.1
				1.5	0.5–4.6

Beaver Dam Eye Study [237]	1709	Dietary intake in the past	Large drusen	0.4	0.2–0.9
		Dietary intake at baseline	Large drusen	0.8	0.4–1.7

Rotterdam Study [235]	4170	Dietary intake	Early and late ARM	0.92 (Hazard Ratio)	0.84–1.0

**Table 11:** Studies investigating the relationship between vitamin A and risk for ARM.

Study	No. of cases	Measure of risk factor	Type of ARM	Odds ratio or relative risk	95% confidence interval
Cross-Sectional Studies					

National Health and Nutrition Examination Survey I [54]	3082	Dietary intake	Early and late ARM	0.74	0.50–1.10

Pathologies Oculaires Liees a L'Age [29, 236]	2157	Plasma Level	Late ARM	0.6	0.17–2.12

Prospective Studies					

Blue Mountains Eye Study [233]	2900	Dietary intake	Early ARM	1.2	0.7–2.0
			Late ARM	1.2	0.5–3.3

Blue Mountains Eye Study [226, 233]	1989	Dietary intake	Early ARM	0.9	0.6–1.6

**Table 12:** Studies investigating the relationship between dietary and serum levels of carotenoids and risk for ARM.

Study	No. of cases	Measure of risk factor	Type of ARM	Odds ratio or relative risk	95% confidence interval
Cross-Sectional Studies					

National Health and Nutrition Examination Survey III [245]	8222	Dietary intake of lutein and zeaxanthin	Pigmentary abnormalities	0.1 (Younger age groups)	0.1–0.3
		Serum level of lutein and zeaxanthin	Early ARM	1.0	0.6–1.5

Prospective Studies					

Beaver Dam Eye Study [237]	1709	Dietary intake of pro-vitamin A carotenoids	Large drusen	0.53	0.3–1.0
				0.45	0.2–1.0

Blue Mountains Eye Study [226, 242]	2900	Dietary intake of carotene	Early ARM	0.7	0.4–1.1
			Late ARM	0.7	0.3–2.0

Blue Mountains Eye Study [226, 242]	1989	Dietary intake of *α*- and *β*-carotene	Early ARM	1.3 (*α*-carotene)	0.8–2.2
				1.2 (*β*-carotene)	0.7–2.1

Rotterdam Study [235]	4170	Dietary intake of *β*-carotene in combination with vitamin C, vitamin E and zinc	Early and late ARM	0.65	0.46–0.92

Pathologies Oculaires Liees a L'Age [246]	899	Plasma Levels of zeaxanthin	Early and Late ARM	0.07	0.01–0.58
		Combined plasma level of lutein and zeaxanthin	Early and late ARM	0.21	0.05–0.79

**Table 13:** Studies investigating the relationship between zinc and risk for ARM.

Study	No. of cases	Measure of risk factor	Type of ARM	Odds ratio or relative risk	95% confidence interval
Cross-Sectional Studies					

Beaver Dam Eye Study [234]	1968	Dietary intake	Early ARM	0.6	0.4–1.0

Beaver Dam Eye Study [234, 237]	1709	Dietary intake	Early ARM	0.7	0.5–1.0
			Late ARM	1.1	0.3–4.1

Blue Mountains Eye Study [233]	2900	Dietary intake	Early ARM	0.8	0.5–1.3
			Late ARM	1.0	0.4–2.8

Blue Mountains Eye Study [226, 233]	1989	Dietary intake	Early ARM	1.3	0.7–2.7

Rotterdam Study [235]	4170	Dietary intake	Early and late ARM	0.91	0.83–0.98

**Table 14:** Studies investigating the relationship between dietary fat/fish intake and risk for ARM.

Study	No. of cases	Measure of risk factor	Type of ARM	Odds ratio or relative risk	95% confidence interval
Cross-Sectional Studies					

National Health and Nutrition Examination Survey III [56]	7883	Total fat intake (percentage of total energy)	Early ARM	1.4	0.9–2.2
			Late ARM	0.7	0.2–2.6 (>60 years of age)

Blue Mountains Eye Study [253]	2900	Energy-adjusted intake of cholesterol	Late ARM	2.71	0.93–7.96
		Frequency of fish consumption per week	Late ARM	0.52	0.22–1.24 (1/wk)
				0.47	0.20–1.11 (2–4/wk)
				0.46	0.12–1.68 (>5/wk)

Prospective Studies					

Beaver Dam Eye Study [254]	2152	Saturated fat %kJ	Early ARM	1.8	1.2–2.7
		Cholesterol Mg/4200 kJ	Early ARM	1.6	1.1–2.4

Blue Mountains Eye Study [255]	2335	Total dietary fat	Early ARM	0.92	0.53–1.59
			Late ARM	1.19	0.36–3.94
		Frequency of fish consumption per week	Early ARM	0.58	0.37–0.90 (1/wk)
			Late ARM	0.25	0.06–1.00 (3/wk)

**Table 15:** Studies investigating the relationship between cumulative sunlight exposure and risk for ARM.

Study	No. of cases	Measure of risk factor	Type of ARM	Odds ratio or relative risk	95% confidence interval
Cross-Sectional Studies					

Beaver Dam Eye Study [260]	4926	Amount of time spent outdoors in summer	Retinal pigmentary changes	1.44	1.01–2.04 (men)
		Amount of leisure time spent outdoors in summer	Late ARM	2.19	1.12–4.25 (men)

Pathologies Oculaires Liees a L'Age [262]	2584	Ambient solar Radiation	Early ARM	0.73	0.54–0.98
		Leisure exposure to sunlight	Early ARM	0.80	0.64–1.00

Prospective Studies					

Beaver Dam Eye Study [261]	3684	Amount of leisure time spent outdoors aged 13–19 years and aged 30–39 years	Early ARM	2.09	1.19–3.65

Beaver Dam Eye Study [258]	2764	Amount of leisure time spent outdoors aged 13–19 years, aged 30–39 years and at baseline examination	Early ARM	2.20	1.02–4.73
		Average annual UV-B exposure	Early ARM	0.83	0.62–1.10
			Late ARM	1.00	0.54–1.82

**Table 16:** Studies investigating the relationship between refractive error and risk for ARM.

Study	No. of cases	Measure of risk factor	Type of ARM	Odds ratio or relative risk	95% confidence interval
Cross-Sectional Studies					

Blue Mountains Eye Study [263]	3654	Each diopter of increase in mean spherical equivalent	Early ARM	1.1	1.0–1.2
			Late ARM	1.0	0.9–1.1

Rotterdam Study [264]	6209	Each diopter of increase towards hypermetropia	Early ARM	1.09	1.04–1.14
			Late ARM	1.09	1.01–1.19

Beijing Eye Study [37]	4439	Hypermetropia	Early ARM	N/A	1.1–1.34
			Late ARM		0.76–1.05

Prospective Studies					

Blue Mountains Eye Study [265]	2335	Each diopter of increase in mean spherical equivalent	Early ARM	1.1	0.98–1.15
			Late ARM	1.1	0.9–1.2

Beaver Dam Eye Study [266]	3684	Hypermetropia +1.00 diopter or more	Early ARM	0.86	0.62–1.19 (Right Eye)
				0.75	0.55–1.03 (Left Eye)
			Late ARM	2.09	0.30–14.49 (Right Eye)
				3.56	0.59–21.74 (Left Eye)

Beaver Dam Eye Study [266]	3306	Hypermetropia +1.00 diopter or more	Early ARM	0.9	0.7–1.1
			Late ARM	1.2	0.6–2.3

Rotterdam Study [264]	4935	Each diopter of increase towards hypermetropia	Early and late ARM	1.05	1.01–1.10

**Table 17:** Studies investigating the relationship between iris color and risk for ARM.

Study	No. of cases	Measure of risk factor	Type of ARM	Odds ratio or relative risk	95% confidence interval
Cross-Sectional Studies					

Copenhagen City Eye Study [269]	924	Brown versus blue iris color	Drusen and decreased vision	0.7	N/A

Blue Mountains Eye Study [270]	3654	Blue versus brown iris colour	Early ARM	1.45	1.1–1.9
			Late ARM	1.69	1.0–2.9
			Early ARM	1.06	0.76–1.47 (Right eye)
				1.15	0.85–1.56 (Left eye)

Prospective Studies					

Beaver Dam Eye Study [271]	3684	Brown iris Color	Late ARM	1.61	0.60–4.33 (Right eye)
				0.99	0.41–2.37 (Left eye)

Beaver Dam Eye Study [271]	2764	Blue versus brown iris color	Soft indistinct drusen	1.53	1.19–1.97
			Retinal pigment abnormalities	0.58	0.41–0.82

**Table 18:** Studies investigating the relationship between cataract and risk for ARM.

Study	No. of cases	Measure of risk factor	Type of ARM	Odds ratio or relative risk	95% confidence interval
Cross-Sectional Studies					

Framingham Eye Study [52, 53]		Nuclear sclerosis	Early and late ARM	<1.00 (All ages)	N/A
		Cortical lens opacity	Early and late ARM	>1.00 (Age 52–74)	N/A

National Health and Nutrition Examination Survey I [54]	3087	Opacity without visual impairment	ARM	1.80	1.40–2.30
		Cataract	ARM	1.14	0.84–1.55

Blue Mountains Eye Study [272]	3654	Nuclear Sclerosis	Early ARM	1.0	0.6–1.9
			Late ARM	1.6	0.5–5.3
		Cortical lens Opacity	Early ARM	1.4	0.9–2.2
			Late ARM	1.7	0.7–4.2
		Posterior Subcapsular lens Opacity	Early ARM	1.1	0.4–2.7
			Late ARM	3.0	1.0–9.3

Beaver Dam Eye Study [273]	4926	Nuclear sclerosis	Early ARM	1.96	1.28–3.01
			Late ARM	1.38	0.52–3.63

ProspectiveStudies					

Beaver Dam Eye Study [181, 273, 274]	2764	Any lens opacity	Early ARM	1.30	1.04–1.63

Copenhagen City Eye Study [38–40]	359	Any lens opacity	Early and late ARM	2.8	1.2–6.2

**Table 19:** Studies investigating the relationship between cataract surgery and risk for ARM.

Study	No. of cases	Measure of risk factor	Type of ARM	Odds ratio or relative risk	95% confidence interval
Cross-Sectional Studies					

Salisbury Eye Evaluation Project + Proyecto VER + Baltimore Eye Survey (Pooled data) [277]	2520 + 4774 + 4396	Previous cataract surgery	Late ARM	1.7	1.1–2.6

Andhra Pradesh Eye Study [41]	3723	Previous cataract surgery	Early and late ARM	3.79	2.1–6.78

Prospective Studies					

Beaver Dam Eye Study [278]	3684	Previous cataract surgery	Late ARM	2.80	1.03–7.63

Beaver Dam Eye Study [278]	2764	Previous cataract surgery	Early ARM	1.36	0.82–2.23
			Late ARM	3.81	1.89–7.69

Blue Mountains Eye Study + Beaver Dam Eye Study (Pooled data) [279]	3684 + 2335	Previous cataract surgery	Late ARM	5.7	2.4–13.6

Blue Mountains Eye Study [280]	1952	Previous cataract surgery	Late ARM	3.3	1.1–9.9

**Table 20:** Studies investigating the relationship between alcohol and risk for ARM.

Study	No. of cases	Measure of risk factor	Type of ARM	Odds ratio or relative risk	95% confidence interval
Cross-Sectional Studies					

Beaver Dam Eye Study [285]	4926	Consumption of beer in the past year	Pigmentary changes	1.13	1.02
			Neovascular AMD	1.41	1.41

Blue Mountains Eye Study [286]	3654	Consumption of spirits	Early ARM	1.61	1.07–2.41
		Consumption of beer	Early ARM	1.42	0.86–2.35
			Late ARM	0.89	0.32–2.48

National Health and Nutrition Examination Survey 1 [287]	3072	Consumption of wine	Early and Late ARM	0.81	0.67–0.99

Los Angeles Latino Eye Study [114]	5875	“Heavy” consumption of alcohol (>5 drinks per session)	Geographic atrophy	12.7	1.5–104.6
			Neovascular AMD	5.8	1.3–25.8

Andhra Pradesh Eye Study [41]	3723	“Light” alcohol drinkers versus non-drinkers	Early and late ARM	0.38	0.19–0.76

Prospective Studies					

Beaver Dam Eye Study [116, 288]	3534	Consumption of >78 g/wk of alcohol from beer versus 0 g/week beer consumption	Early ARM	N/A	N/A [Power 0.08 (10.6% Incidence versus 6.9% Incidence)]

Reykjavik Eye Study [34, 35]	846	Current alcohol consumption	Soft drusen	0.48	0.28–0.82 (<Monthly)
				0.34	0.16–0.72 (>Monthly)

Copenhagen City Eye Study [38, 39]	359	Alcohol consumption >250 g/wk	Early ARM	4.6	1.1–19.2
